# Epigenome-wide DNA methylation analysis of late-stage mild cognitive impairment

**DOI:** 10.3389/fcell.2024.1276288

**Published:** 2024-01-16

**Authors:** Yi Zhang, Shasha Shen

**Affiliations:** Institute of Neuroscience, Panzhihua University, Panzhihua, China

**Keywords:** EWAS, LMCI, methylation, blood, Alzheimer’s disease

## Abstract

**Background:** Patients with late-stage mild cognitive impairment (LMCI) have a higher risk of progression to Alzheimer’s disease (AD) than those with early-stage mild cognitive impairment (EMCI). However, previous studies have often pooled EMCI and LMCI patients into a single MCI group, with limited independent investigation into the pathogenesis of LMCI.

**Methods:** In this study, we employed whole-genome methylation association analysis to determine the differences in peripheral blood methylation profiles between 663 cognitive aging (CN) and 554 LMCI patients.

**Results:** Our results revealed 2,333 differentially methylated probes (DMPs) and 85 differentially methylated regions (DMRs) specific to LMCI. The top hit methylation sites or regions were associated with genes such as SNED1, histone deacetylases coding gene HDACs, and HOX and ZNF gene family. The DNA methylations upregulated the expression of HDAC4, HDAC8, and HOX family genes HOXC5 and HOXC9, but they downregulated the expression of SNED1, ADCYAP1, and ZNF family genes ZNF415 and ZNF502. Gene Ontology (GO) and KEGG analysis showed that the genes associated with these methylation sites were predominantly related to the processes of addiction disorders, neurotransmission, and neurogenesis. Out of the 554 LMCI patients included in this study, 358 subjects (65%) had progressed to AD. Further association analysis between the LMCI subjects with a stable course (sLMCI) and those who progressed to AD (pLMCI) indicated that the methylation signal intensities of HDAC6, ZNF502, HOXC5, HOXC6, and HOXD8 were associated with increased susceptibility to AD. Protective effects against progression to AD were noticed when the methylation of SNED1 and ZNF727 appeared in LMCI patients.

**Conclusion:** Our findings highlight a substantial number of LMCI-specific methylated biomarkers that differ from those identified in previous MCI case–control studies. These biomarkers have the potential to contribute to a better understanding of the pathogenesis of LMCI.

## 1 Introduction

Mild cognitive impairment (MCI) is a complex and heterogeneous condition between normal cognitive aging (CN) and dementia, specifically Alzheimer’s disease (AD) (Petersen et al., 2001; McGirr et al., 2022). Patients with MCI have memory complaints and objective memory impairment that is abnormal for their age, while their general cognitive function remains relatively preserved, enabling them to perform everyday activities independently (Petersen, 2004; Chen et al., 2022). MCI can be subcategorized into early-stage MCI (EMCI) and late-stage MCI (LMCI), where LMCI is accompanied by more severe memory decline in cognitive domains, such as language, executive function, and visuospatial skills (Aisen et al., 2010; Zhang et al., 2019). It has been reported that approximately 10%–15% of patients each year, MCI progresses to AD, and 75% of such individuals have LMCI (Petersen et al., 2001; Farias et al., 2009; Jessen et al., 2014; Tábuas-Pereira et al., 2016). Therefore, the early recognition of MCI, especially LMCI, is essential for preventing AD.

Epigenetic changes in the central nervous system (CNS) and peripheral blood have widely been used for the early diagnosis of MCI and AD (Lunnon et al., 2014; Madrid et al., 2018; Roubroeks et al., 2020; Vasanthakumar et al., 2020; Li et al., 2021). These changes reflect potential immune system disorders, altered proteostasis, neuronal decay, and changes in brain structure that are associated with the disease (Lunnon et al., 2014; Madrid et al., 2018; Roubroeks et al., 2020; Vasanthakumar et al., 2020; Li et al., 2021). However, most studies have pooled patients with EMCI and LMCI into a single MCI group, which may obscure the different disease progression risks between these two subgroups (Zhang et al., 2019; Vasanthakumar et al., 2020; Li et al., 2021). Given that the risk of conversion to AD is higher for LMCI than for EMCI (36% vs. 15%) (Jessen et al., 2014), identifying epigenetic biomarkers specific to LMCI can be more beneficial in reducing the incidence of AD and improving the effectiveness of rehabilitation exercises and medication. In this study, we compared the peripheral blood methylome of CN individuals and LMCI patients. We revealed a significant number of LMCI-specific methylated biomarkers, which differ from those identified in previous MCI case–control studies. These biomarkers may help to elucidate the pathogenesis of LMCI.

## 2 Materials and methods

### 2.1 Subjects

The data utilized in this study were sourced from the Alzheimer’s Disease Neuroimaging Initiative (ADNI) database. The ADNI is a multicenter, longitudinal study encompassing approximately 50 sites across the United States and Canada, and it was initiated in 2003 with the primary aim of monitoring the progression of AD through the use of clinical and cognitive assessments, magnetic resonance imaging (MRI), fludeoxyglucose positron emission tomography (PET), amyloid PET, cerebrospinal fluid analysis, and blood biomarker analysis. For the purposes of ADNI research, a total of 1,720 samples from 653 individuals who participated in two phases of ADNI (ADNI2 and ADNIGO) were selected for DNA methylation analysis. These samples were randomized using a modified incomplete balanced block design, in which all of the samples from a single subject were placed on the same chip, while the remaining space on the chip was filled with age-matched samples from a subject of the opposite sex with a different diagnosis.

Amnestic MCI was defined in accordance with the diagnostic criteria established by ADNI as detailed in the ADNI protocol (http://adni.loni.usc.edu/methods/documents/). Specifically, the criteria were as follows: a) a score of 24–30 on the Mini-Mental State Examination (MMSE); b) a self-reported memory complaint, as well as objective evidence of memory loss as measured by education-adjusted scores on the Wechsler Memory Scale Logical Memory II; c) a Clinical Dementia Rating (CDR) score of 0.5; and d) the absence of significant impairment in other cognitive domains, as well as the preservation of activities of daily living and the absence of dementia (Jack Jr et al., 2008). MCI was further classified into two subtypes, namely, EMCI and LMCI based on the severity of memory impairment. The criteria for LMCI were the same as those for EMCI, with the exception that the memory impairment on the Logical Memory II subscale had to be more severe. Specifically, the cutoff scores for LMCI were ≤8 for individuals with 16 or more years of education, ≤4 for 8–15 years of education, and ≤2 for 0–7 years of education. The corresponding cutoff scores for EMCI were 9–11 for individuals with 16 or more years of education, 5–9 for 8–15 years of education, and 3–6 for 0–7 years of education (Jack Jr et al., 2008).

The datasets utilized in this study included clinical information and epigenetic data obtained from the ADNI database (http://adni.loni.usc.edu), accessed on 12 June 2021. The methylation profile pertained to 1,220 samples, including 665 individuals with CN status and 555 individuals with LMCI status. Data processing and quality control procedures were performed on the collected data, which resulted in the selection of 663 CN and 554 LMCI samples for downstream analysis.

### 2.2 Data quality control

The analysis was conducted in accordance with the previously outlined protocol (Fortin et al., 2017; Tian et al., 2017). Specifically, we employed a rigorous quality control and preprocessing approach utilizing the Minfi package from the R software. The detection *p* values (detP) were calculated through the “m + u” method, which compared the total DNA signal (methylated + unmethylated) for each probe to the background signal level. None of the samples had mean detP value higher than 0.05, but three samples were excluded due to a low ratio of unmethylated to methylated sites (uMeth/mMeth), i.e., less than 10.5 (as shown in Supplementary Figure S1). The call rate was determined as the proportion of probes present in each sample. The probes with a detection *p*-value of 0.05 or higher in at least 1% of the samples were filtered out. Finally, a total of 1,217 samples (663 CN and 554 LMCI) comprising 823605 probes were retained for downstream analysis.

### 2.3 Identification of differentially methylated probes (DMPs)

We performed a probe-wise analysis to identify DMPs using the Bioconductor package limma. To ensure statistical validity, beta values were converted to M-values, which are considered more statistically robust than beta values due to their higher detection rates and true positive rates for both highly methylated and unmethylated CpG sites. The experimental design was modeled as follows: ≈class (disease status) + age + gender + education + DNA source (buffy coat or whole blood) + B cells + CD4 T + CD8 T + Mono + Neu + NK, where the last six terms represent cell type composition estimations obtained using estimateCellCounts from R Package FlowSorted.Blood.EPIC at default settings. The estimateCellCounts function combined the reference library from FlowSorted.Blood.450K with the target methylation dataset to build the model with cellular deconvolution algorithms for the relative quantification of the proportion of cell types (Houseman et al., 2012; Fortin et al., 2017; Tian et al., 2017). Because the study was prone to significant inflation and bias of test statistics, we applied a Bayesian method based on estimation of the empirical null distribution in the Bioconductor package limma to control for inflation of test statistics and for lambda inflation factors. A stringent threshold using Bonferroni correction was used to declare study-wide significance (adjusted *p*-value <0.05).

### 2.4 Identification and annotation of differentially methylated regions (DMRs)

We employed a DMR analysis in the R package DMRcate to identify a group of CpGs associated with LMCI. DMRcate models Gaussian kernel smoothing within a predefined distance (1 kbp in this study) and collapses contiguous significant CpGs (*p* < 0.05) after multiple testing correction. The default algorithm parameters were utilized, which included the following: a) regions with gaps ≥1,000 nucleotides between significant CpG sites were separated; b) regions containing at least two different CpGs within 1 kb with a minimum methylation difference of 10% were included in the analysis. The regions with an adjusted *p*-value lower than 0.05 from Stouffer’s, Harmonic, and Fisher’s tests were considered to be significant. Visualization and functional analysis of DMRs were performed by means of the R package coMET.

### 2.5 Functional analysis of DMPs

Using the missMethyl R package, we performed a generalized gene set enrichment analysis to assess pathway enrichment through a hypergeometric test, which took into account the number of CpG sites per gene on the EPIC array. The analysis included curated gene sets from the KEGG database and Gene Ontology (GO) gene sets related to biological processes, cellular components, and molecular functions. The pathways or terms with a Benjamini–Hochberg false discovery rate (FDR)–corrected *p*-value lower than 0.05 were considered significant. The ratio values of the number of significantly annotated genes in a particular pathway to the total number of genes in the pathway were calculated.

### 2.6 Gene expression profile

We utilized the microarray expression data of 318 samples (207 CN, 175 LMCI) in the ADNI cohort to investigate the effect of DNA methylation on the overlapping genes. A total of 28 protein-coding genes (PCGs) overlapping between DMPs or DMRs were included. We processed the raw data based on the standard quality control (QC) procedures described in ADNI (http://adni.loni.usc.edu/methods/documents/). The raw expression values were normalized for differential gene expression (DEG) analysis with the Bioconductor package limma. The model design was similar to the previously described DMP analysis. Specifically, we adjusted for the effect of age, gender, education, DNA source, and cell type compositions. The genes with a Benjamini–Hochberg FDR-corrected *p*-value lower than 0.05 were considered to be DEGs.

### 2.7 Serum proteomic profiling

We further employed the serum proteomic profile data of 20 samples (10 with CN, 10 with LMCI) in the ADNI cohort to validate the results of epigenome-wide association studies (EWAS). The data were obtained from the Gene Expression Omnibus (GEO) under accession number GSE74763. Due to the limitation of fluorescence probes for specific proteins, we could only filter out the proteomic data of HDAC4, HDAC6, HDAC8, HOXC5, HOXC6, HOXC9, ZNF415, and ZNF502. The raw data were processed and normalized in line with Invitrogen’s standard instructions (www.invitrogen.com/protoarray). One-way ANOVA was used for statistical analysis. Proteins with a Benjamini–Hochberg FDR-corrected *p*-value lower than 0.05 were considered to be differentially expressed across the groups.

### 2.8 Association analysis between DMPs and conversion from LMCI to AD

A logistic regression model was built to evaluate the effects of 27 candidate methylation probes on the conversion from LMCI to AD. These DMPs were associated with SNED1, RP11-526P5.2, ADCYAP1, HDACs, and HOX and ZNF gene family (listed in Figure 6; Supplementary Table S12). A total of 554 LMCI subjects, including 196 subjects with a stable course (sLMCI) and 358 subjects who had progressed to AD (pLMCI), were involved in the analysis. The effects of age, gender, education, DNA source, and ApoEε4 alleles were adjusted for in the model. We calculated the odds ratio (OR) and confidence interval of each DMP to assess the effect of DNA methylation on the progression to AD. OR values with *p*-value lower than 0.05 were considered significant.

Furthermore, we filtered pLMCI subjects and evaluated the association of DMPs with the progression time and cognitive impairment levels. We checked the Pearson correlation coefficients between DMP signal intensity and indicators related with cognitive impairment, such as the scores at the baseline diagnosis with the mini-mental state examination (MMSE), the clinical dementia rating scale sum of boxes (CDRSB), the modified preclinical Alzheimer cognitive composite using digit symbol substitution test (mPACCdigit), and the modified preclinical Alzheimer cognitive composite using trail-making test part B (mPACCtrailsB). Higher MMSE, mPACCdigit, and mPACCtrailsB scores indicate better cognitive function. However, a higher CDRSB score represents more severe cognitive impairment. Correlation coefficients with a *p*-value lower than 0.05 were considered significant.

Besides, we measured the speed of cognitive decline based on MMSE (MMSE_speed), CDRSB (CDRSB_speed), mPACCdigit (mPACCdigit_speed), and mPACCtrailsB (mPACCtrailsB_speed). The speed scores were calculated as |Score _(first diagnosis as AD)_–Score _(baseline diagnosis as LMCI)_|/progression time _(months)_. Higher MMSE_speed, CDRSB_speed, mPACCdigit_speed, and mPACCtrailsB_speed scores represent greater speeds of cognitive decline. We also calculated the Pearson correlation coefficients between DMP signal intensity and scores of cognitive decline speed. Correlation coefficients with a *p*-value lower than 0.05 were considered significant.

## 3 Results

### 3.1 Study participants

The association of DNA methylation with LMCI was analyzed by using the Illumina EPIC array datasets from the ADNI. We filtered three samples that had been lost during processing or excluded during the QC procedure, and we finally kept 1,217 samples for peripheral blood DNA methylation analysis (Table 1; Supplementary Figure S1; Supplementary Table S1). The demographic characteristics and cognitive assessments of the samples used in the comparative analysis are presented in Table 1.

**TABLE 1:** Demographic data of the selected ADNI subjects separated by diagnosis group.

Variable	CN	LMCI	*p*-value
N	663	554	
Age	74.77 ± 5.46	73.07 ± 7.22	3.35E^-06^
Education	16.45 ± 2.65	16.01 ± 2.87	5.20E^-03^
Gender (proportion of males)	50.53%	61.55%	9.99E^-01^
Gender (proportion of females)	49.47%	38.45%	
ApoEε4 (proportion of subjects with 0 alleles)	73.30%	45.13%	9.12E^-01^
ApoEε4 (proportion of subjects with 1 alleles)	24.74%	43.68%	
ApoEε4 (proportion of subjects with 2 alleles)	1.96%	11.19%	

ApoEε4, the ε4 allele of the Apolipoprotein E gene; CN, cognitive normal; EMCI, early mild cognitive impairment; LMCI, early mild cognitive impairment; AD, Alzheimer’s disease. Data were expressed as mean ± standard error of the mean (SEM). One-way ANOVA, was used for statistical analysis of age and education across groups. Chi-square test was used for statistical analysis of gender and ApoEε4 allele across groups.

### 3.2 Alterations of blood cell composition in different groups

**FIGURE 1:**
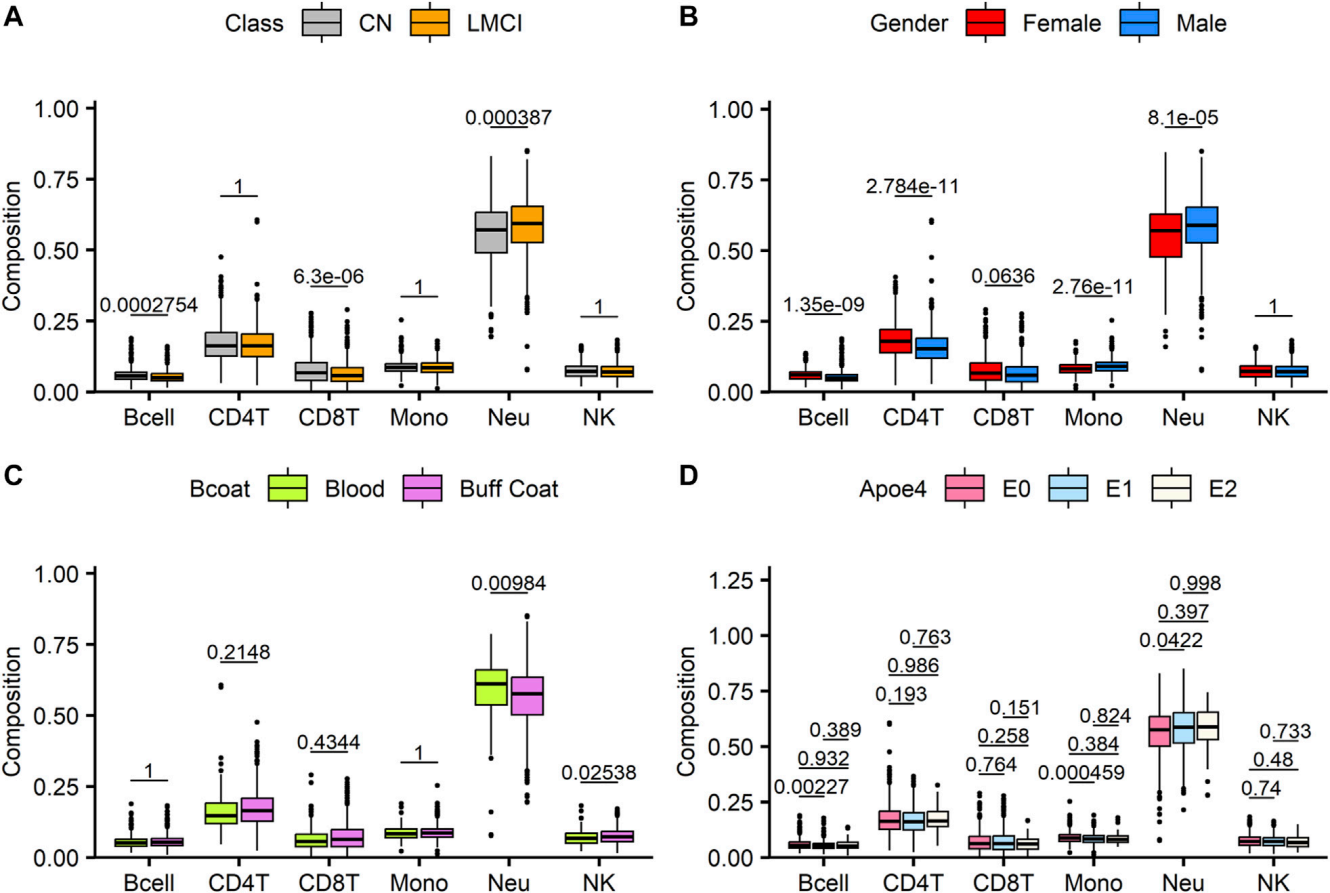
Analysis of estimated blood cell type composition in late-stage mild cognitive impairment (LMCI) versus normal cognitive aging individuals (CN). Abundance of specific blood cell types was estimated based on unique methylation markers for cell identity. Estimated proportions of B lymphocytes (Bcell), CD4T cells (CD4T), CD8T cells (CD8T), monocytes (mono), neutrophils (Neu) and natural killer cells (NK) were compared across disease groups **(A)**, genders **(B)**, sample sources **(C)** and Apoe4 alleles **(D)**. Significant differences across groups are estimated by using Wilcoxon test after correction for multiple observations **(A–C)** or one-way analysis of variance with Bonferroni correction for multiple observations **(D)**.

Altered blood cell composition has been observed in various neurodegenerative disorders, thus suggesting the possibility of systemic immune perturbations. DNA methylation signals offer a promising approach for estimating the relative abundance of different lymphocyte subpopulations. Compared with the CN cases, the patients with LMCI presented a smaller estimated proportion of B cells and CD8 T cells (*p* = 2.75E^-04^ and *p* = 6.3E^-06^, respectively, *t*-test with Wilcoxon *post hoc* test), a higher proportion of neutrophils (*p* = 3.87E^-04^), and no significant changes in CD4 T cells, monocytes, and natural killer cells (NK) (*p* > 0.05) (Figure 1A). We also evaluated the changes in blood cell composition driven by sex distribution (Figure 1B) and DNA sources (buff coat or whole blood; Figure 1C). Except for CD8 T cells and NK cells, the overall blood composition varied between the male and female groups, where the female cases showed an increased proportion of B cells and CD4 T cells (*p* = 1.35E^-09^ and *p* = 2.78E^-11^, respectively, *t*-test with Wilcoxon *post hoc* test), but a reduced proportion of monocytes and neutrophils (*p* = 2.76E^-11^ and *p* = 8.1E^-05^, respectively). Previous studies have reported that differences in the storage of the sample used for DNA isolation (buff coat or whole blood) influence the cell composition. However, in our study, the whole-blood samples only demonstrated significant alterations in neutrophils and NK cells compared with the buff-coat samples (Figure 1C), showing increased neutrophils (*p* = 9.84E^-03^) and reduced NK cells (*p* = 2.54E^-02^). Moreover, we assessed the effect of APOE4 gene alleles on blood lymphocyte composition. There were significant differences in lymphocyte composition only between individuals with zero alleles and those with one allele (*p* < 0.05; Figure 1D).

### 3.3 DMPs in LMCI vs. CN

**FIGURE 2:**
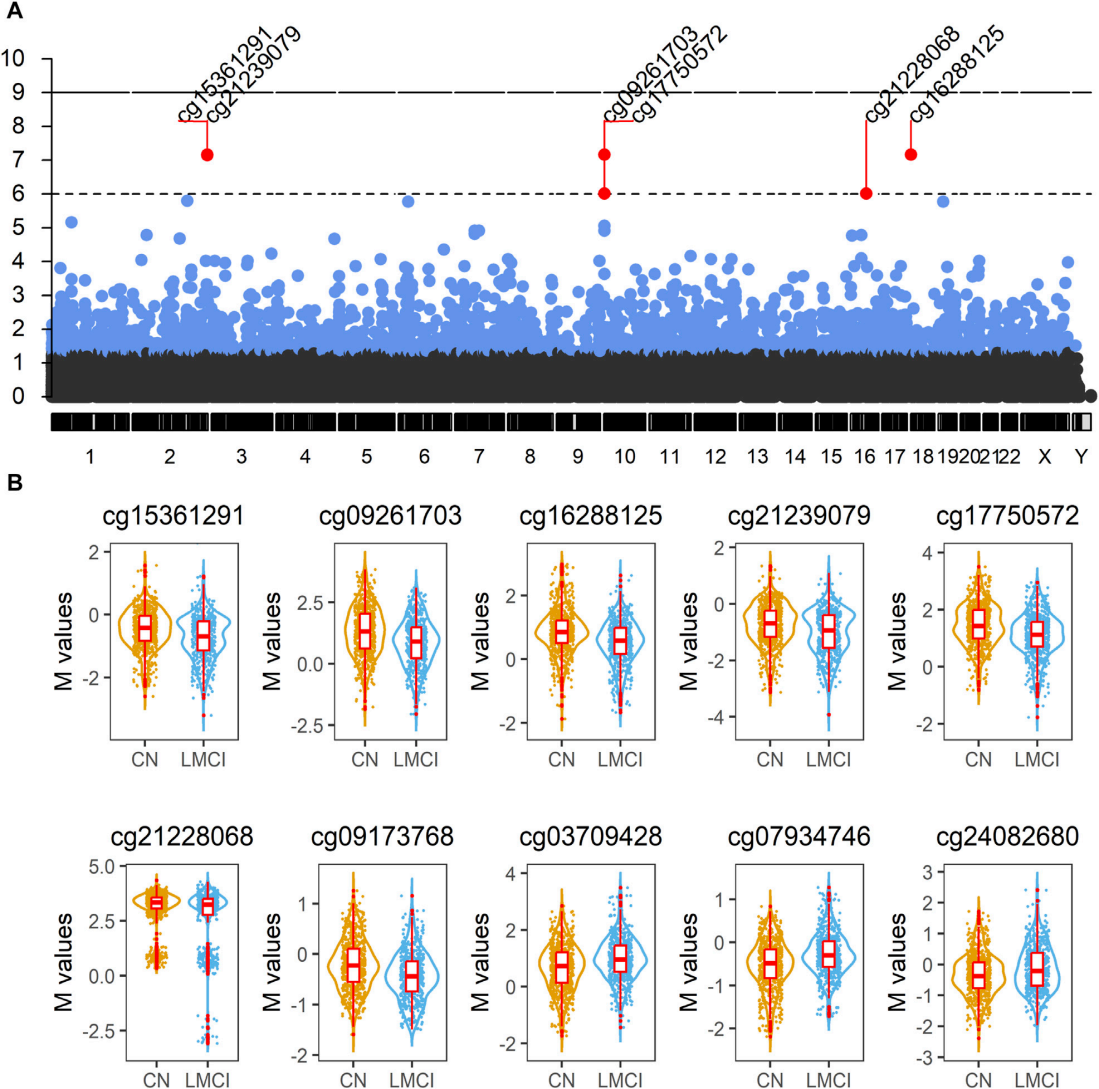
Analysis of blood methylation in late-stage mild cognitive impairment (LMCI) versus normal cognitive aging individuals (CN). **(A)** Manhattan plot compiling genome-wide methylation sites for the comparison of LMCI versus CN. Differentially methylated probes (DMPs) above short dashed line showed genome-wide significance (adjusted *p* ≤ 1E^−06^). **(B)** Representative violin plots of select top 10 significant DMPs showing increased or decreased methylation in LMCI cases compared to CN cases.

A cross-sectional analysis of blood methylation was performed in LMCI and CN cases. Linear regression models were employed, adjusting for age, gender, education, DNA source, and blood cell composition. We identified 2,333 DMPs in LMCI vs. CN (raw *p* < 1.42 E^-06^; adjusted *p* < 0.05), 709 of which reached genome-wide significance at adjusted *p* < 0.01 (raw *p* < 8.56E^-06^; Table 2; Figure 2; Supplementary Table S2). The Quantile–Quantile plot showed that the genomic inflation factor (lambda) was less than 1.10 (lambda = 1.0115; Figure 2; Supplementary Figure S2). Overall changes in methylation were modest, with |log2 of fold-change| ≤ 0.8 (Table 2; Supplementary Table S2). Among these DMPs, 1,608 CpG sites showed increased methylation in the LMCI patients (625 of them without overlapping annotated genes, e.g., cg03709428 and cg07934746; Table 2; Supplementary Table S2), while the rest showed lower levels of methylation in the LMCI cases compared with the CN group (Supplementary Table S2).

**TABLE 2:** List of differentially methylated probes (DMPs) with adjusted *p* less than Bonferroni correction threshold of 0.05.

Probes	Chr	Pos	Strand	GencodeCompV12	LogFC	Ave M-value	t	*p*-value	Adjusted *p*-value
cg15361291	Chr2	242,003,523	−	AC005237.4; SNED1	−0.28	−0.58	−7.48	1.45E^-13^	6.89E^-08^
cg09261703	Chr10	2,543,967	+	RP11-526P5.2	−0.41	1.10	−7.42	2.16E^-13^	6.89E^-08^
cg16288125	Chr18	904,243	+	ADCYAP1	−0.30	0.71	−7.40	2.51E^-13^	6.89E^-08^
cg21239079	Chr2	242,003,549	−	SNED1; AC005237.4	−0.32	−0.86	−7.36	3.47E^-13^	7.14E^-08^
cg17750572	Chr10	2,544,120	+	RP11-526P5.2	−0.29	1.29	−6.95	5.86E^-12^	9.63E^-07^
cg21228068	Chr16	50,827,518	+	CYLD; RP11-327F22.4	−0.48	2.89	−6.93	7.02E^-12^	9.63E^-07^
cg09173768	Chr2	176,989,349	−	HOXD9; HOXD-AS2	−0.19	−0.32	−6.83	1.35E^-11^	1.59E^-06^
cg03709428	Chr6	31,275,741	+		0.31	0.82	6.79	1.72E^-11^	1.68E^-06^
cg07934746	Chr19	15,774,266	+		0.21	−0.41	6.78	1.84E^-11^	1.68E^-06^
cg24082680	Chr1	63,249,199	+	ATG4C	0.26	−0.25	6.55	8.44E^-11^	6.95E^-06^

Chr, chromosome; Pos, DNA, base position; Strand, DNA, strand; GencodeCompV12, GENCODE, Comprehensive database version 12 containing all transcripts at protein-coding loci; LogFC, log2 of fold change of M-value across groups; Ave M-value, average M–value across all samples.

We found 74 sex-linked DMPs, 2 DMPs with unknown chromosome location, and 2,257 DMPs uniformly distributed across the autosomal chromosomes. Six of the 10 most significant CpGs were associated with PCGs (Table 2; Supplementary Table S4), including DMPs annotated to SNED1 (cg15361291, chr2: 242,003,523, adjusted *p* = 6.89E^-08^; cg21239079, chr2: 242,003,549, adjusted *p* = 7.14E^-08^), ADCYAP1 (cg16288125, chr18: 904,243, adjusted *p* = 6.89E^-08^), CYLD (cg21228068, chr16: 50,827,518, adjusted *p* = 9.63E^-07^), HOXD9 (cg09173768, chr2: 176,989,349, adjusted *p* = 1.59E^-06^), and ATG4C (cg24082680, chr1: 63,249,199, adjusted *p* = 6.95E^-06^). Four non PCGs (NCGs; Table 2; Supplementary Table S4), namely, AC005237.4 (cg15361291, chr2: 242,003,523, adjusted *p* = 6.89E^-08^; cg21239079, chr2: 242,003,549, adjusted *p* = 7.14E^-08^), RP11-526P5.2 (cg09261703, chr10: 2,543,967, adjusted *p* = 6.89E^-08^; cg17750572, chr10: 2,544,120, adjusted *p* = 9.63E^-07^), RP11-327F22.4 (cg21228068, chr16: 50,827,518, adjusted *p* = 9.63E^-07^), and HOXD-AS2 (cg09173768, chr2: 176,989,349, adjusted *p* = 1.59E^-06^) were annotated by six of the top 10 DMPs. The most significant site was cg15361291, located in chr2: 242,003,523, which showed 21% lower methylation (variation = |1−2^−logfc^|×100%) in the LMCI subjects than in the CN individuals (Table 2). The second CpG site (cg09261703), located in Chr10: 2,543,967, had 33% lower methylation (variation = |1−2^−logfc^|×100%) in the subjects with LMCI than in the CN participants (Table 2).

### 3.4 DMR analysis

**FIGURE 3:**
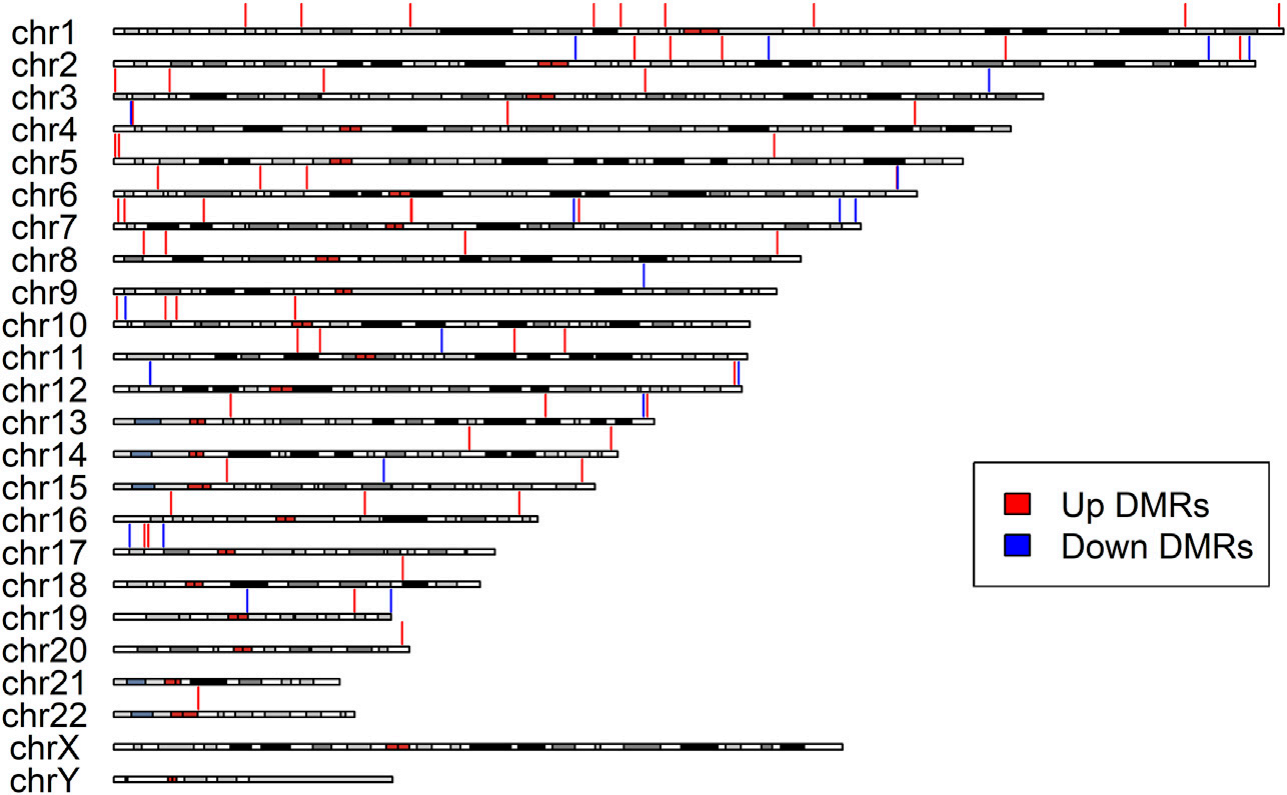
Differentially Methylated Regions (DMRs) distribution along the chromosomes. The red vertical lines indicate upregulated DMRs, while the blue vertical lines indicate downregulated DMRs. All identified DMRs are localized within autosomes; no DMRs were detected within sex chromosomes.

DMR analysis enabled identification of the regions in the genome that showed concerted changes in methylation and were deemed to have a large impact on modulating transcription. Overall, the DMRcate algorithm identified 85 DMRs as significantly associated with cognitive decline in the participants with LMCI (Table 3; Supplementary Table S3). All of the DMRs in the genome were located in autosomal chromosomes (Figure 3). Among them, we identified 46 DMRs annotated to PCGs (Supplementary Table S5), such as DMRs annotated to SNED1 [chr2: 242,002,695 to 242,003,549 (4 probes), Fisher-corrected *p* = 1.58E^-12^], ZNF727 [chr7: 63,505,584 to 63,506,261 (9 probes), Fisher-corrected *p* = 2.34E^-10^], COL5A2 [chr2: 190,043,537 to 190,044,983 (8 probes), Fisher-corrected *p* = 3.01E^-10^], NTNG1 [chr1: 108,022,767 to 108,023,486 (7 probes), Fisher-corrected *p* = 5.72E^-10^], and CREBZF [chr11: 85,393,571 to 85,394,069 (6 probes), Fisher-corrected *p* = 3.73E^-09^]. Recent studies have underlined the potential involvement of these genes in regulating immune cell function and inflammation, as well as their potential implication in the pathogenesis of various neurological disorders, such as AD and Parkinson disease (Naba et al., 2014; Cassandri et al., 2017; Krushkal et al., 2020; Barqué et al., 2021; Bu et al., 2021; Vallet et al., 2021; Arunachalam et al., 2022; Raouf Issa et al., 2022). The methylation region associated with the crucial gene TTC23, which plays a vital role in protein QC during brain development (Roubroeks et al., 2020; Vasanthakumar et al., 2020; Li et al., 2021; Lee et al., 2022), had the second highest density of significant CpG probes (13 probes; Supplementary Table S3; Supplementary Figure S4).

**TABLE 3:** List of differentially methylated regions (DMRs) ranked by Fisher’s multiple comparison statistics.

Chr	Start	End	Width	No. DMPs	Min. FDR	Stouffer	HMFDR	Fisher	Mean. diff	Overlapping genes
Chr10	2,543,474	2,544,596	1,123	8	1.30E^-46^	1.13E^-18^	5.07E^-07^	4.33E^-21^	−0.0345	RP11-526P5.2
Chr2	242,002,695	242,003,549	855	4	6.73E^-28^	9.65E^-10^	1.40E^-07^	1.58E^-12^	−0.0288	SNED1; AC005237.4
Chr7	63,505,584	63,506,261	678	9	1.57E^-30^	4.87E^-09^	6.13E^-05^	2.34E^-10^	0.0252	ZNF727; RP11-3N2.13
Chr2	190,043,537	190,044,983	1,447	8	1.78E^-28^	6.38E^-05^	6.75E^-04^	3.01E^-10^	0.0145	COL5A2
Chr1	108,022,767	108,023,486	720	7	2.20E^-26^	1.86E^-09^	1.52E^-03^	5.72E^-10^	0.0262	NTNG1
Chr11	85,393,571	85,394,069	499	6	7.68E^-24^	3.49E^-10^	1.32E^-03^	3.73E^-09^	0.0308	CREBZF
Chr5	1,102,675	1,104,195	1,521	6	1.19E^-18^	5.78E^-09^	1.39E^-03^	5.84E^-08^	0.0126	SLC12A7
Chr1	63,249,197	63,249,765	569	9	1.06E^-26^	3.79E^-02^	6.02E^-05^	6.43E^-08^	0.0163	
Chr16	53,543,684	53,544,321	638	5	2.44E^-18^	1.90E^-08^	6.85E^-04^	1.33E^-07^	0.0201	
Chr15	99,789,622	99,791,336	1715	13	3.04E^-22^	3.90E^-07^	3.25E^-03^	2.59E^-07^	0.0183	TTC23

Chr, chromosome; Start, start base position of region; End, end base position of region; Width, width of region; No. DMPs, number of DMPs, within the region; Min. FDR, the minimum adjusted *p* from the CpGs constituting the significant region; Stouffer, the adjusted *p* of Stouffer’s test; HMFDR, the adjusted *p* of Harmonic test; Fisher, the adjusted *p* of Fisher’s test; Mean. diff, the mean methylation difference across groups in Fisher’s test.

Moreover, DMRcate detected 12 DMRs annotated to NCGs (Supplementary Table S5), such as DMRs annotated to AC005237.4 [chr2: 242,002,695 to 242,003,549 (4 probes), Fisher-corrected *p* = 1.58E^-12^], RP11-3N2.13 [chr7: 63,505,584 to 63,506,261 (9 probes), Fisher-corrected *p* = 2.34E^-10^], LINC00116 [chr2: 110,969,641 to 110,970,909 (8 probes), Fisher-corrected *p* = 3.21E^-07^], CTC-281F24.1 [chr17: 6,557,720 to 6,559,109 (7 probes), Fisher-corrected *p* = 3.75E^-06^], and MIR4520A [chr17: 6,557,720 to 6,559,109 (7 probes), Fisher-corrected *p* = 3.75E^-06^]. The most significant DMR identified in this study that was associated with conversion status in the patients with LMCI was annotated to RP11-526P5.2 [chr10: 2,543,474 to 2,544,596 (8 probes), Fisher-corrected *p* = 4.33E^-21^; Table 3; Supplementary Table S3]. Six DMPs in this region, including the second significant CpG-site cg09261703, were highly correlated and located in the upstream CpG island of the RP11-526P5.2 gene (Supplementary Figure S4).

### 3.5 Methylation profiles in HOX and ZNF family genes

Some of the top hit DMPs and DMRs were closely associated with HOX and ZNF family genes. We summarize the significant methylation sites of HOX and ZNF family genes in Tables 4, 5; Supplementary Table S6; Supplementary Figure S5. The results showed that nine DMPs, such as cg09173768 (chr2: 176,989,349, adjusted *p* = 1.59E^-06^) and cg15410411 (chr2: 54,392,884, adjusted *p* = 3.59E^-04^), were enriched in the gene regions of HOXC4, HOXC5, HOXC6, HOXC9, HOXC-AS1, HOXD3, HOXD8, HOXD9, HOXD-AS1 and HOXD-AS2. However, no significant DMRs were found in HOX family genes. In the case of ZNF family genes, 31 methylation sites, such as cg13947469 (chr7: 63,505,871, adjusted *p* = 1.53E^-05^) and cg14768256 (chr3: 44,754,587, adjusted *p* = 1.09E^-04^), were shown to be significant in the LMCI patients. Two DMRs overlapping with ZNF727 or ZNF502 were associated with LMCI (adjusted *p* < 0.05).

**TABLE 4:** List of differentially methylated probes (DMPs) related with HOX family genes.

Probes	Chr	Pos	Strand	GencodeCompV12	LogFC	Ave M-value	t	*p*-value	Adjusted *p*-value
cg09173768	Chr2	176,989,349	−	HOXD9; HOXD-AS2	−0.19	−0.32	−6.83	1.35E^-11^	1.59E^-06^
cg15410411	Chr12	54,392,884	+	HOXC9; HOXC-AS1; HOXC5; HOXC6	−0.19	−1.16	−5.56	3.30E^-08^	3.59E^-04^
cg21336435	Chr12	54,398,561	−	HOXC5; HOXC6	−0.14	−0.96	−4.91	1.04E^-06^	2.90E^-03^
cg08254359	Chr12	54,398,518	−	HOXC5; HOXC6	−0.15	−0.59	−4.62	4.32E^-06^	6.41E^-03^
cg05611263	Chr12	54,425,634	−	HOXC4; HOXC5	−0.12	1.30	−4.28	2.00E^-05^	1.66E^-02^
cg06316886	Chr2	177,027,043	−	HOXD3	−0.13	−1.82	−4.23	2.47E^-05^	1.86E^-02^
cg22934308	Chr2	177,038,617	−	HOXD3; HOXD-AS1	0.14	−0.22	4.22	2.65E^-05^	1.94E^-02^
cg07783843	Chr2	176,997,311	+	HOXD-AS2; HOXD8	0.10	−0.56	3.91	9.65E^-05^	4.04E^-02^
cg14324370	Chr2	177,042,789	−	HOXD-AS1; AC009336.24	0.08	−4.23	3.88	1.09E^-04^	4.29E^-02^

Chr, chromosome; Pos, DNA, base position; Strand, DNA, strand; GencodeCompV12, GENCODE, Comprehensive database version 12 containing all transcripts at protein-coding loci; LogFC, log2 of fold change of M-value across groups; Ave M-value, average M–value across all samples.

**TABLE 5:** List of top 10 differentially methylated probes (DMPs) related with ZNF family genes.

Probes	Chr	Pos	Strand	GencodeCompV12	LogFC	Ave M-value	t	*p*-value	Adjusted *p*-value
cg13947469	Chr7	63,505,871	−	ZNF727; RP11-3N2.13	0.27	−1.43	6.36	2.78E^-10^	1.53E^-05^
cg14768256	Chr3	44,754,587	+	ZNF502	0.25	1.06	5.90	4.82E^-09^	1.09E^-04^
cg06088684	Chr2	180,610,608	−	ZNF385B	−0.13	2.79	−5.41	7.44E^-08^	5.83E^-04^
cg18831899	Chr17	5,019,056	−	ZNF232; USP6	0.16	2.03	5.33	1.17E^-07^	7.57E^-04^
cg09560297	Chr19	37,406,349	−	ZNF568; ZNF829	0.14	0.94	5.11	3.65E^-07^	1.52E^-03^
cg05769153	Chr19	53,636,398	+	ZNF415	0.20	1.75	5.04	5.36E^-07^	1.89E^-03^
cg05223766	Chr19	53,590,304	+	ZNF160	0.15	−2.07	4.86	1.33E^-06^	3.36E^-03^
cg01511534	Chr16	3,284,640	−	ZNF200	0.14	−1.72	4.80	1.81E^-06^	4.02E^-03^
cg16428517	Chr16	3,317,428	+	ZNF263	0.12	2.51	4.71	2.75E^-06^	4.91E^-03^
cg05241461	Chr19	22,816,980	−	ZNF492	0.15	−3.48	4.71	2.82E^-06^	4.99E^-03^

Chr, chromosome; Pos, DNA, base position; Strand, DNA, strand; GencodeCompV12, GENCODE, Comprehensive database version 12 containing all transcripts at protein-coding loci; LogFC, log2 of fold change of M-value across groups; Ave M-value, average M–value across all samples.

### 3.6 DNA methylation in genes associated with histone modification

To investigate the DNA methylation status of genes that encode the enzymes of histone modification, we summarized the DMPs and DMRs related to histone acetyltransferases (HATs), histone deacetylases (HDACs), histone methyltransferases (HMTs), histone demethylases (KDMs), protein kinases (PTKs), and protein phosphatases (PPs) in Table 6; Supplementary Figure S6. Unfortunately, we did not find any DMPs in HATs, HMTs, KDMs, PTKs, and PPs. Only five DMPs in HDAC4, HDAC6, or HDAC8, such as cg14865678 (chr2: 239,984,042, adjusted *p* = 1.71E^-02^) and cg20784693 (chr2: 239,984,030, adjusted *p* = 1.94E^-02^), were identified. There were no DMRs in the region overlapping HMTs, HDACs, KDMs, PTKs, and PPs.

**TABLE 6:** List of differentially methylated probes (DMPs) related with HDAC family genes.

Probes	Chr	Pos	Strand	GencodeCompV12	LogFC	Ave M-value	t	*p*-value	Adjusted *p*-value
cg14865678	Chr2	239,984,042	+	HDAC4	0.13	0.83	4.27	2.10E^-05^	1.71E^-02^
cg20784693	Chr2	239,984,030	+	HDAC4	0.15	0.55	4.22	2.67E^-05^	1.94E^-02^
cg04067339	ChrX	71,760,492	−	HDAC8	0.09	−1.28	3.94	8.76E^-05^	3.85E^-02^
cg24616736	ChrX	48.659,713	−	HDAC6	0.10	−1.84	3.92	9.24E^-05^	3.95E^-02^
cg09155776	Chr2	239,984,105	−	HDAC4	0.11	−0.22	3.90	1.03E^-04^	4.18E^-04^

Chr, chromosome; Pos, DNA, base position; Strand, DNA, strand; GencodeCompV12, GENCODE, Comprehensive database version 12 containing all transcripts at protein-coding loci; LogFC, log2 of fold change of M-value across groups; Ave M-value, average M–value across all samples.

### 3.7 Enriched pathways related to neurotransmission

**FIGURE 4:**
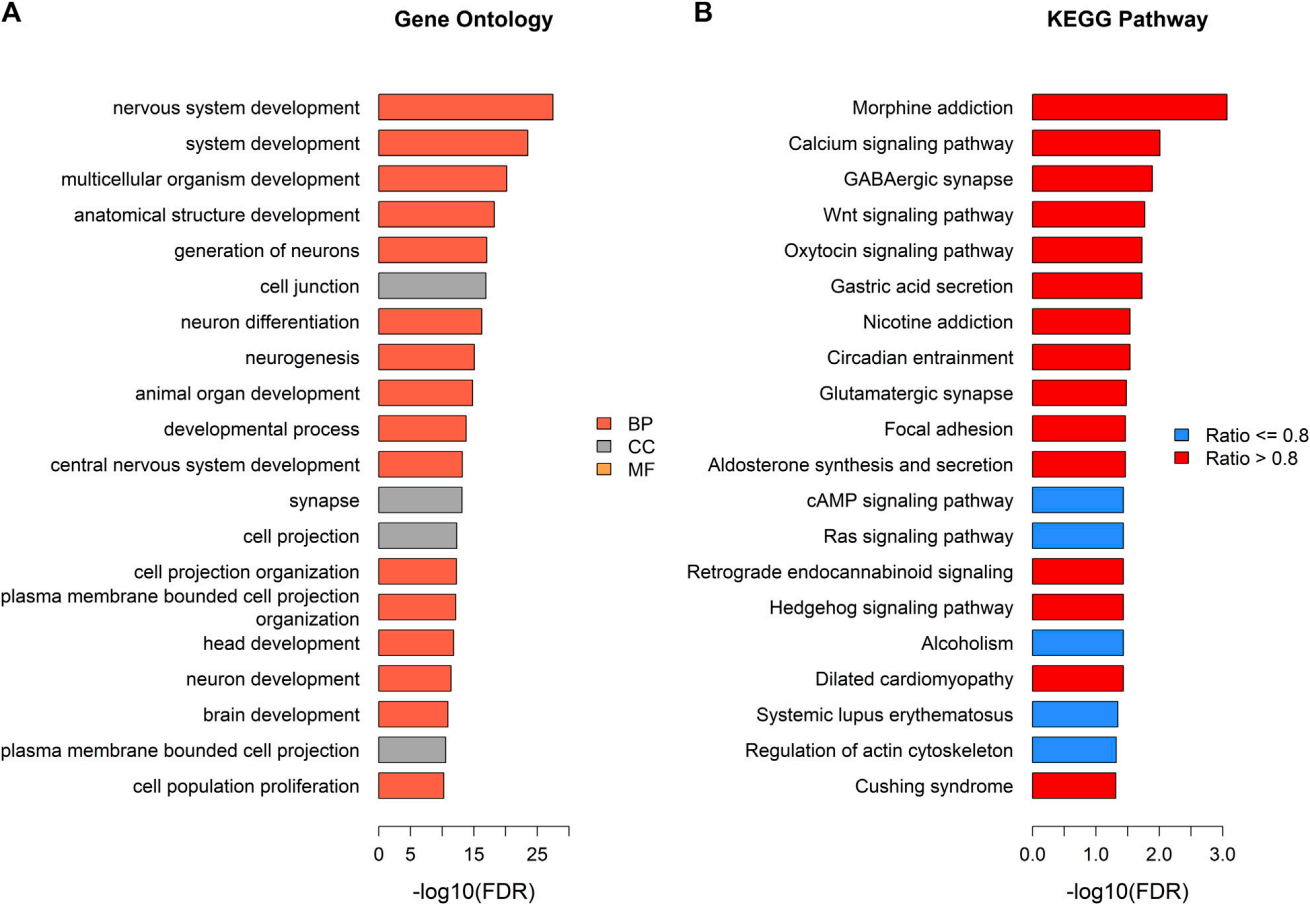
Pathway enrichment analysis of DMPs annotated genes. **(A)** Top gene ontology (GO) enrichment terms with adjusted *p* < 0.05 (tomato color bars: BP; grey color bars: CC; orange color bars: MF). **(B)** Top KEGG pathways (blue color bars: pathways with ratio of genes annotated by DMPs ≤ 0.8; red color bars: pathways with ratio of genes annotated by DMPs >0.8).

Generalized gene set enrichment analysis with the hypergeometric test in the R package missMethyl was performed to gain biological insight from these epigenetic differences. GO terms and KEGG pathways with adjusted *p* values less than 0.05 were selected to annotate the PCGs of differential CpG sites (Supplementary Table S4). This selection yielded 503 GO terms (Supplementary Table S7), including 357 terms of biological processes (BP), 75 terms of cell components (CC), and 71 terms of molecular functions (MF), and 20 KEGG pathways (Supplementary Table S8). A total of 157 of the identified GO terms reached enrichment significance at an adjusted *p* < 0.0001 (99 BP, 28 CC, and 30 MF; Supplementary Table S7). The results of GO analysis showed that the DMPs annotated genes were involved in nervous system development, neurogenesis, and cell (neuron) projection pathways (adjusted *p* < 0.05, ratio values of DMPs annotated genes in the pathways ranging between 0.67 and 1; Figure 4; Supplementary Tables S4, S7). Parallel testing in the KEGG gene sets showed a marked enrichment in addiction disorders and neurotransmission, such as morphine addiction, the calcium signaling pathway, and GABAergic synapses (adjusted *p* < 0.05, ratio values of DMPs annotated genes in the pathways ranging between 0.73 and 0.92; Figure 4; Supplementary Tables S4, S8).

### 3.8 Influence of DNA methylation on gene expression

To investigate the influence of DNA methylation on the gene expression, we examined the expression levels of 28 PCGs that overlapped with DMPs or DMRs. In general, these target genes exhibited low expression abundance, that is, their average expression counts were lower than 50 (Table 7; Supplementary Table S9). As shown in Table 7, a total of 11 genes were significantly differentially expressed between the LMCI and CN individuals. Four of the DEGs were HOX or ZNF family genes, namely, HOXC9 (adjusted *p* = 5.49E^-03^), HOXC5 (adjusted *p* = 1.15E^-02^), ZNF415 (adjusted *p* = 7.17E^-05^), and ZNF502 (adjusted *p* = 7.17E^-05^). In addition, we found that the expression of HDAC8 (adjusted *p* = 8.75E^-03^) and HDAC4 (adjusted *p* = 1.03E^-02^) was significantly upregulated in the LMCI patients. In contrast, SNED1 and ADCYAP1, which were annotated by the top hit DMPs and DMRs, were downregulated in the LMCI patients.

**TABLE 7:** Gene expression validation of candidate DMPs or DMRs related protein-coding genes (11 significant genes).

Genes	LogFC	AveExpr	t	*p*-value	Adjusted *p*-value
NTNG1	−1.34	2.31	−9.80	2.45E^-20^	6.87E^-19^
COL5A2	1.41	2.48	9.04	8.75E^-18^	1.23E^-16^
ADCYAP1	−1.06	2.72	−7.09	6.71E^-12^	6.26E^-11^
ZNF415	−0.75	2.63	−4.47	1.02E^-05^	7.17E^-05^
CYLD	−1.48	7.55	−3.63	3.23E^-04^	1.81E^-03^
HOXC9	0.71	3.58	3.27	1.18E^-03^	5.49E^-03^
HDAC8	0.71	4.43	3.08	2.19E^-03^	8.75E^-03^
HDAC4	0.79	4.19	2.99	2.94E^-03^	1.03E^-02^
HOXC5	0.64	4.15	2.92	3.70E^-03^	1.15E^-02^
SNED1	−0.57	3.98	−2.45	1.47E^-02^	4.12E^-02^
ZNF502	−0.72	5.25	−2.38	1.80E^-02^	4.57E^-02^

LogFC, log2 of fold change of expression counts across groups; AveExpr, the average value of log2 expression count.

### 3.9 Validation with proteomic profiling

**FIGURE 5:**
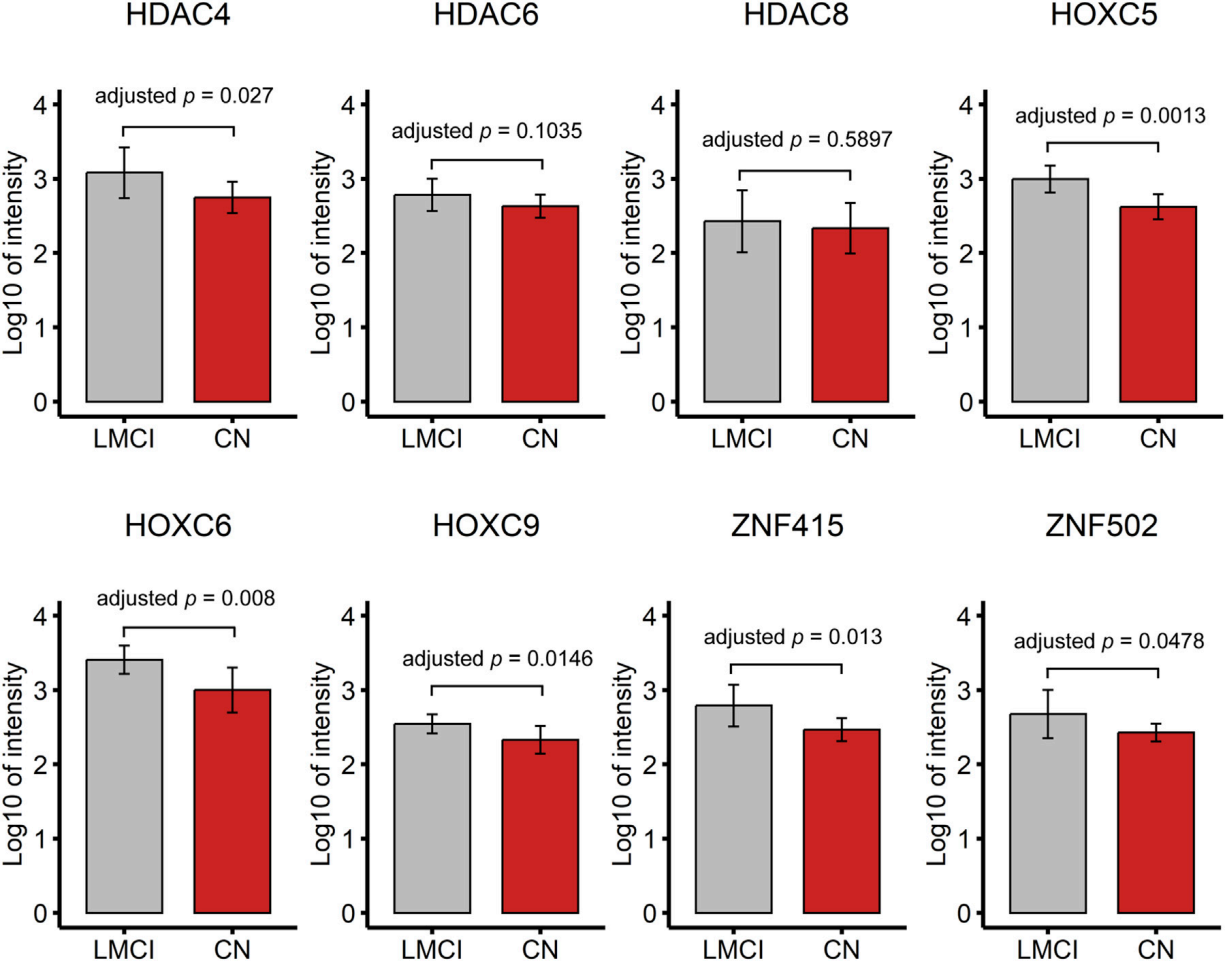
Proteomic profile of candidate DMPs or DMRs related proteins (8 candidate proteins).

The serum proteomic profile analysis of the eight proteins associated with DMPs further validated the EWAS results. The results showed that six of these proteins, namely, HDAC4, HOXC5, HOXC6, HOXC9, ZNF415, and ZNF502, were significantly differentially expressed between LMCI and CN (adjusted *p* < 0.05; Figure 5; Supplementary Table S10). Consistent with the results of the gene expression profile analysis, the expression of proteins HDAC4 (adjusted *p* = 2.70E^-02^), HOXC5 (adjusted *p* = 1.30E^-03^), and HOXC9 (adjusted *p* = 1.46E^-02^) was significantly upregulated in the LMCI patients (Figure 5; Supplementary Table S10). However, the proteomic results of proteins ZNF415 and ZNF502 were opposite those of the results of the gene expression profile analysis. Both of ZNF415 and ZNF502 were also significantly upregulated in the LMCI patients (adjusted *p* < 0.05; Table 7; Figure 5; Supplementary Tables S9, S10).

### 3.10 Association of DNA methylation level with progression to AD

**FIGURE 6:**
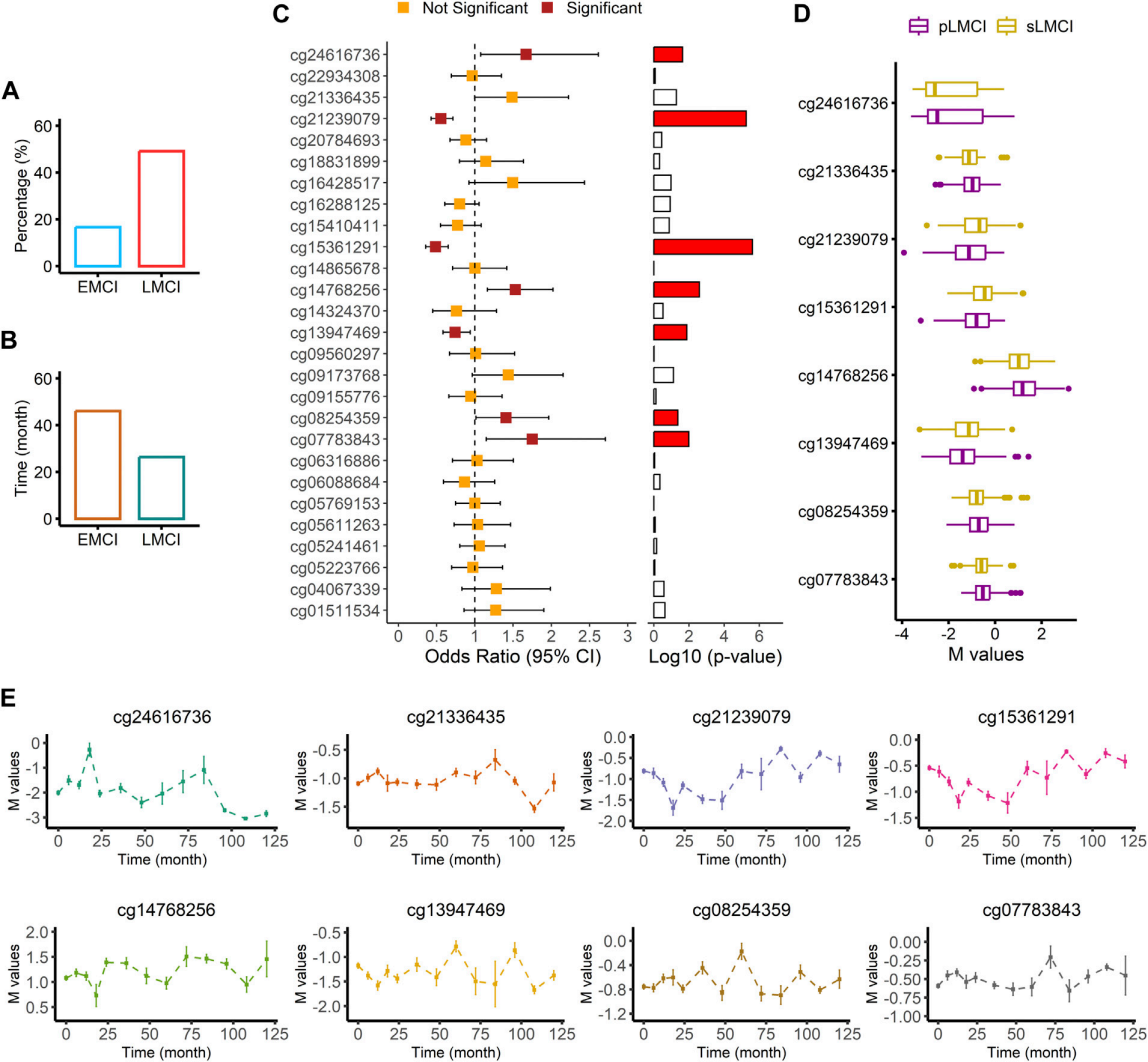
Association analysis of AD progression from LMCI. **(A)** Comparison of progression probability between EMCI and LMCI. **(B)** Comparison of progression time between EMCI and LMCI. **(C)** Odds ratio values from logistic regression with comparison between sLMCI and pLMCI. **(D)** Box plot of methylation signal intensity across groups. **(E)** Distribution curve of methylation signal intensity over time.

We screened the samples (756 with EMCI and 1,120 with LMCI) in the ADNI cohort to calculate the proportion of MCI progression to AD. As shown in Figure 6A; Supplementary Table S11, we found that the probability of LMCI progressing to AD was about three times higher than that of EMCI (49% vs. 17%). The average progression speed of the LMCI patients was much faster than that of the EMCI patients (26 months vs. 46 months; Figure 6B; Supplementary Table S11). Out of the 554 LMCI patients included in this study, 358 subjects (65%) had progressed to AD. The OR values from the logistic regression model indicated that HDAC6-associated DMP cg24616736 [OR = 1.67, 95% CI (1.08–2.62), *p* = 2.33E^-02^], ZNF502-associated DMP cg14768256 [OR = 1.53, 95% CI (1.16–2.02), *p* = 2.57E^-03^], HOXC5- and HOXC6-associated DMP cg08254359 [OR = 1.41, 95% CI (1.01–1.97), *p* = 4.30E^-02^], and HOXD8-associated DMP cg07783843 [OR = 1.75, 95% CI (1.15–2.71), *p* = 1.04E^-02^] were associated with increased susceptibility to AD in LMCI subjects (Figures 6C, D; Supplementary Table S12). SNED1-associated DMPs cg15361291 [OR = 0.48, 95% CI (0.36–0.65), *p* = 2.49E^-06^] and cg21239079 [OR = 0.55, 95% CI (0.43–0.71), *p* = 5.53E^-06^] and ZNF727-associated DMP cg13947469 [OR = 0.74, 95% CI (0.58–0.94), *p* = 1.33E^-02^] showed protective associations with the risk of progression to AD from LMCI (Figures 6C, D; Supplementary Table S12). DMP cg21336435 highly correlated with cg08254359 (Supplementary Figure S5), and both of them were associated with the expression of HOXC5 and HOXC6 (Figures 6C, D; Supplementary Table S12). However, the OR of cg21336435 was not significant [OR = 1.49, 95% CI (1.00–2.23), *p* = 5.22E^-02^; Figures 6C, D; Supplementary Table S12].

Two methylation sites, namely, cg24616736 (*r* = −0.26, *p* = 9.73E^-07^) and cg13947469 (*r* = 0.17, *p* = 1.59E^-03^) were significantly associated with progression time from LMCI to AD (Supplementary Table S13). The distribution curve of methylation signal intensity over time clearly showed that subjects with a weaker methylated signal of cg24616736 had slower progression speed (Figure 6E). Progression speed of subjects with a high unmethylated signal of cg24616736 was mainly between 96 and 120 months (Figure 6E). A similar trend was noticed in the distribution of cg21336435 (Figure 6E). Although there was no significant correlation between progression time and signal intensity of protective DMPs cg21239079 and cg15361291 (*p* > 0.05), we found that subjects with a high methylated signal needed more time for progression from LMCI to AD (Figure 6E). The progression speed in subjects with a high methylated signal of cg21239079 or cg15361291 mainly ranged between 84 and 120 months (Figure 6E).

**FIGURE 7:**
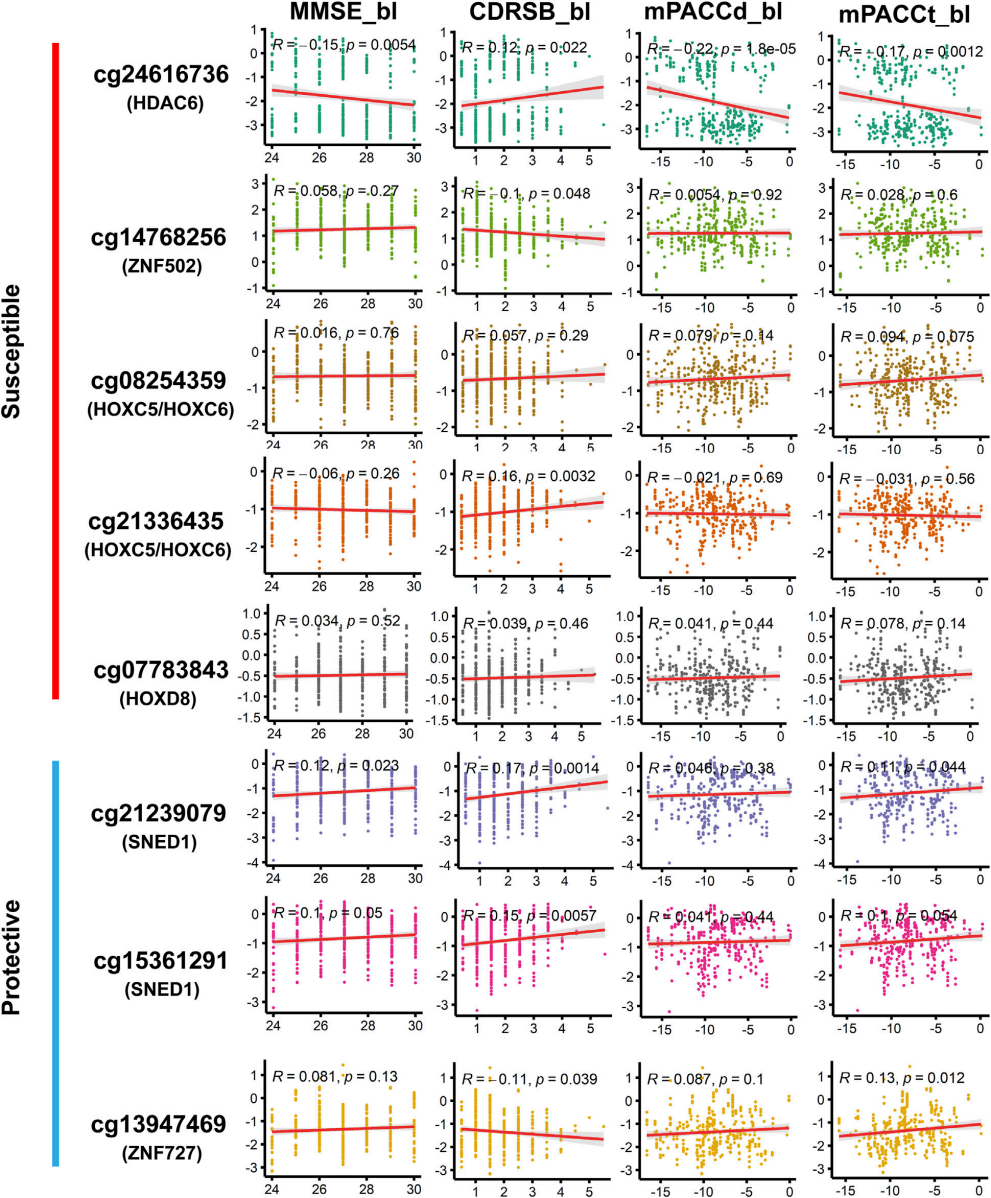
Correlation of methylation signal intensities of DMPs with the cognitive scores at baseline diagnosis. MMSE, mini-mental state examination; CDRSB, clinical dementia rating scale sum of boxes; mPACCdigit, modified preclinical Alzheimer cognitive composite that used digit symbol substitution test; mPACCtrailsB, modified preclinical Alzheimer cognitive composite that used trail-making test part B. Higher scores of MMSE, mPACCdigit, and mPACCtrailsB, represent better cognitive function. However, higher score of CDRSB represent more severe cognitive impairment. Correlation coefficients with *p*-value lower than 0.05 were considered to be significant.

**FIGURE 8:**
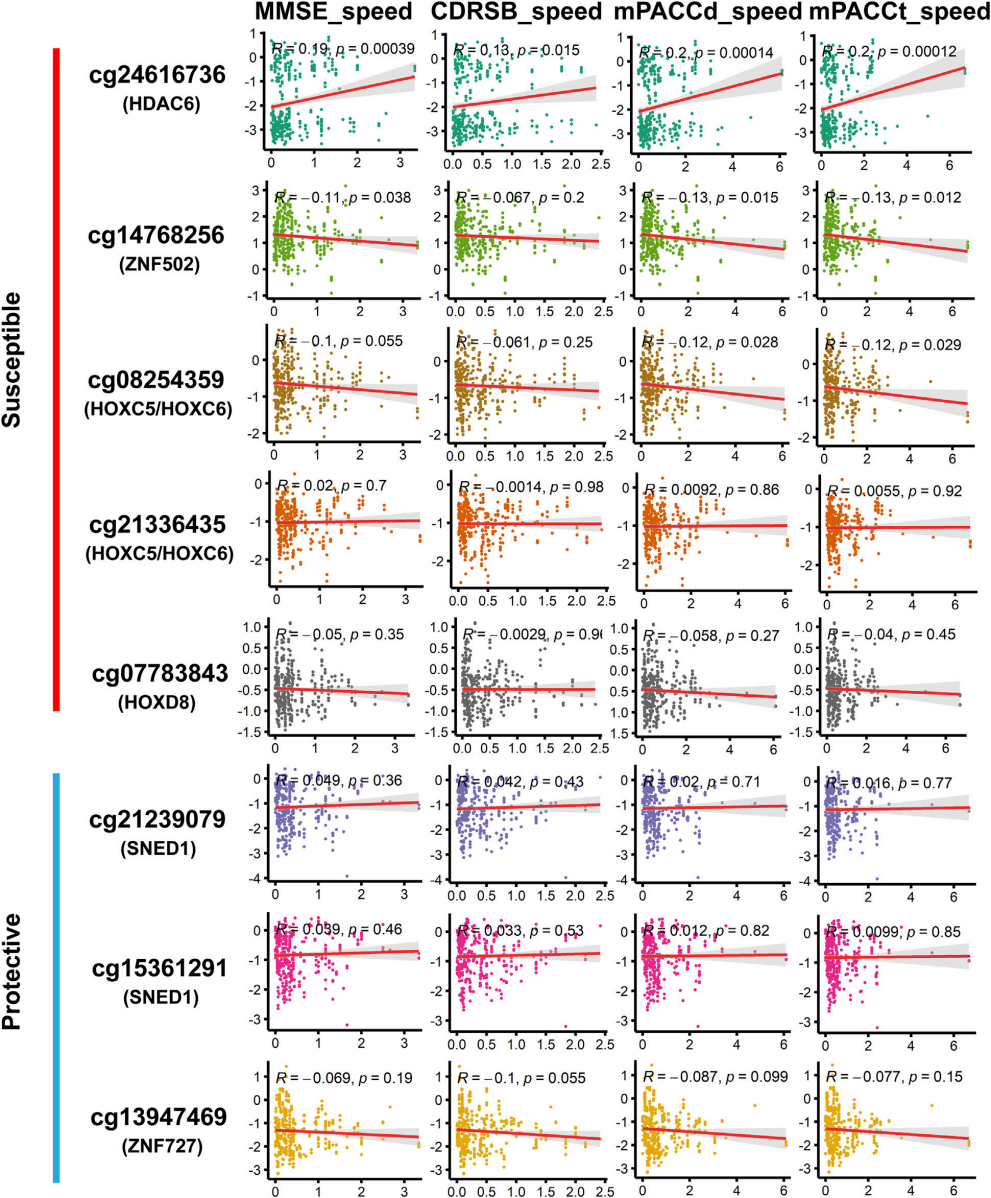
Correlation of methylation signal intensities of DMPs with the speed of cognitive decline. The speed scores were calculated as |Score _(first diagnosis as AD)_–Score _(baseline diagnosis as LMCI)_ |/progression time _(months)_. MMSE_speed: the speed of cognitive decline based on MMSE; CDRSB_speed: the speed of cognitive decline based on CDRSB; mPACCdigit_speed: the speed of cognitive decline based on mPACCdigit; mPACCtrailsB_speed: the speed of cognitive decline based on mPACCtrailsB. Higher scores of MMSE_speed, CDRSB_speed, mPACCdigit_speed, and mPACCtrailsB_speed, represent higher speed of cognitive decline. Correlation coefficients with *p*-value lower than 0.05 were considered to be significant.

Among the DMPs increasing susceptibility to AD, HDAC6-associated DMP cg24616736 was significantly correlated with cognitive scores at baseline diagnosis (Figure 7; Supplementary Table S14), namely, MMSE_bl (*r* = −0.15, *p* = 5.36E^-03^), CDRSB_bl (*r* = 0.12, *p* = 2.20E^-02^), mPACCdigit_bl (*r* = −0.22, *p* = 1.77E^-05^), and mPACCtrailsB_bl (*r* = −0.17, *p* = 1.23E^-03^), and was also significantly correlated with the speed of cognitive score decline (Figure 8; Supplementary Table S14), namely, MMSE_speed (*r* = 0.19, *p* = 3.95E^-04^), CDRSB_speed (*r* = 0.13, *p* = 1.49E^-02^), mPACCdigit_speed (*r* = 0.20, *p* = 1.40E^-04^), and mPACCtrailsB_speed (*r* = 0.20, *p* = 1.20E^-04^). As shown in Figures 7, 8, ZNF502-associated DMP cg14768256 was significantly correlated with MMSE_speed (*r* = −0.11, *p* = 3.79E^-02^), CDRSB_bl (*r* = −0.10, *p* = 4.75E^-02^), mPACCdigit_speed (*r* = −0.13, *p* = 1.45E^-02^), and mPACCtrailsB_speed (*r* = −0.13, *p* = 1.24E^-02^). HOXC5- and HOXC6-associated DMP cg08254359 was significantly correlated with the cognitive decline speed (Figure 8; Supplementary Table S14), namely, mPACCdigit_speed (*r* = −0.12, *p* = 2.76E^-02^) and mPACCtrailsB_speed (*r* = −0.12, *p* = 2.92E^-02^). DMP cg21336435, which was highly correlated with cg08254359, was the only DMP that was significantly correlated with CDRSB_bl (*r* = 0.16, *p* = 3.20E^-03^; Figure 7; Supplementary Table S14). There was no significant correlation between HOXD8-associated DMP cg07783843 and the cognitive scores at baseline diagnosis or the speed of cognitive score decline (*p* > 0.05; Figures 7, 8; Supplementary Table S14). Moreover, we found that the LMCI patients with a higher intensity of cg21239079, which was associated with SNED1, had better cognitive ability as measured by the MMSE_bl (*r* = 0.12, *p* = 2.29E^-02^) and mPACCtrailsB_bl (*r* = 0.11, *p* = 4.37E^-02^) scores, but also had a higher CDRSB_bl score (*r* = 0.17, *p* = 1.37E^-03^), which represents declined cognitive function (Figure 7; Supplementary Table S14). Similarly, cg15361291, another protective DMP associated with SNED1, was positively correlated with both MMSE_bl (*r* = 0.10, *p* = 4.99E^-02^; Figure 7; Supplementary Table S14) and CDRSB_bl (*r* = 0.15, *p* = 5.68E^-03^; Figure 7; Supplementary Table S14). ZNF727-associated DMP cg13947469 was negatively correlated with CDRSB_bl (*r* = −0.11, *p* = 3.93E^-02^; Figure 7; Supplementary Table S14), but positively correlated with mPACCtrailsB_bl (*r* = 0.13, *p* = 1.17E^-02^; Figure 7; Supplementary Table S14).

## 4 Discussion

Both patients with EMCI and LMCI generally exhibit preserved daily activities but present slight cognitive deficits (Grundman et al., 2004; Petersen, 2004; Zhang et al., 2019). Patients with LMCI show more severe impairment in episodic memory than those with EMCI, which has led to the belief that LMCI typically arises during a progression from EMCI (Aisen et al., 2010; Zhang et al., 2019). Previous studies that pooled patients with EMCI and LMCI into a single MCI group have hindered research into the pathogenic mechanisms of LMCI and the elucidation of factors that contribute to LMCI progression to AD (Jessen et al., 2014; Zhang et al., 2019; Vasanthakumar et al., 2020; Chen et al., 2022).

In this study, a total of 2,333 DMPs and 85 DMRs were found in the LMCI patients. The high-risk genes identified in previous EWAS that combined EMCI and LMCI groups into a single MCI group for comparison with CN (Lo et al., 2011; Dumurgier et al., 2017; Chouliaras et al., 2018; Roubroeks et al., 2020; Vasanthakumar et al., 2020), such as FLRT2, were not confirmed to be associated with LMCI in the present study. It is possible that LMCI patients have a higher likelihood of progression to AD than those with EMCI (Jessen et al., 2014); thus, the comparative analysis of LMCI and CN showed more similar results with the studies of AD progression from CN or MCI. Previous EWAS and molecular genetic studies have shown that the DMPs or DMRs associated with HOX and ZNF family genes are closely associated with the onset of AD or progression from MCI to AD (Cassandri et al., 2017; Smith et al., 2018; Roubroeks et al., 2020; Bu et al., 2021; Li et al., 2021; Arunachalam et al., 2022). In this study, gene expression and proteomic profile analysis confirmed that the DNA methylations in LMCI could disrupt the expression of HDAC, HOX, and ZNF family genes. These methylations were closely associated with the cognitive impairment in LMCI patients as measured by the scores of MMSE, CDRSB, mPACCdigit, and mPACCtrailsB.

In the case of HOX family genes, aberrantly expressed HOXB and HOXA genes have been validated as high-risk genes for AD (Smith et al., 2018; Roubroeks et al., 2020; Li et al., 2021; Arunachalam et al., 2022). However, few studies have directly linked HOXC genes to AD or the direct formation of MCI. Only one study has shown that HOX Antisense Intergenic RNA (HOTAIR), transcribed from the antisense strand of the HOXC locus, may be associated with central nervous system inflammation and potentially induce AD (Lu et al., 2022). In this study, we revealed that upregulated expression of HOXC5, HOXC6, and HOXC9 may be associated with the onset of LMCI. Results from EWAS and proteomic profiling showed that increased unmethylated signals of positions such as cg08254359 and cg21336435 could cause high expression levels of HOX family proteins in LMCI patients. These alterations in specific sites of HOX family genes may be related to the cognitive decline in LMCI, and further influence the progression speed from LMCI to AD. While the precise molecular biology links between HOXC genes and LMCI remain unclear, it is apparent that HOX family genes play a crucial role in the occurrence of LMCI.

Peripheral blood EWAS is helpful for identifying the changes in common methylation status across tissues, such as brain and peripheral lymphocytes. This is the reason why the GO and KEGG analyses revealed that the DMPs-associated genes were significantly enriched in the pathways of addiction disorders, neurotransmission, and neurogenesis. The results from peripheral blood EWAS may provide new insights into the link between immune dysfunction and neurodegeneration. Based on DNA methylation signals, we could estimate the composition of lymphocyte subpopulations. We found that the patients with LMCI had lower abundance of B cells and CD8^+^ T cells and higher abundance of neutrophils (Neu) compared with the CN individuals. These findings suggest that LMCI patients exhibit signs of abnormal immune function. Most of the genes associated with DMPs were closely associated with the maintenance of both neural and immune systems. For example, SNED1, which is associated with the top hit DMPs, has been demonstrated to function as a promoter of breast cancer metastasis and amyotrophic lateral sclerosis, and its abnormal expression significantly affects the survival outcome of these patients (Naba et al., 2014; Tarr et al., 2019; Krushkal et al., 2020; Barqué et al., 2021; Vallet et al., 2021). Similarly, the HOX and ZNF family genes have been proven to influence immune function and contribute to the development of neurological system disorders, such as glioblastoma and Parkinson’s disease (Cassandri et al., 2017; Bu et al., 2021; Arunachalam et al., 2022; Raouf Issa et al., 2022).

Investigation in the large population of the ADNI cohort showed that the probability of progressing to AD was about three times higher from LMCI than from EMCI (49% vs. 17%), which is consistent with the findings reported by other research groups (Jessen et al., 2014; Zhang et al., 2019; Vasanthakumar et al., 2020; Chen et al., 2022). Furthermore, we found that the average progression speed of the LMCI patients was much faster than that of the EMCI patients (26 months vs. 46 months). These results demonstrate the importance of independently exploring the pathogenesis of each stage of MCI. Progression analysis indicated that DMPs associated with HDAC6, ZNF502, HOXC5, HOXC6, and HOXD8 were associated with increased susceptibility to AD in LMCI subjects. In contrast, DMPs associated with SNED1 and ZNF727 showed protective associations with the risk of progression from LMCI to AD. In particular, DMP cg24616736, which was associated with HDAC6, showed the strongest correlation with progression time and the speed of cognitive decline in the LMCI patients. We found that both the methylation status and protein expression level of HDAC6 were different between LMCI and CN. This finding suggests that HDAC6 may be a crucial histone deacetylase in the whole process from CN to MCI and further progression to AD. Previous evidence has indicated the important role of HDAC6 in tau-mediated neurodegeneration, and HDAC6 may be involved in various neurodegenerative diseases such as AD, Parkinson disease, amyotrophic lateral sclerosis, and Huntington disease (Zhang et al., 2013; Trzeciakiewicz et al., 2020; Li et al., 2022). However, the epigenetic regulation mechanism behind the expression of HDAC6 is not yet clear. Both LMCI and AD exhibit symptoms of cognitive decline; therefore, targeted inhibition or degradation of HDAC6 as a therapeutic approach for AD could potentially have preventive effects on the occurrence of LMCI.

This study also demonstrated the presence of an association between ZNF family genes and cognitive impairment in LMCI patients. Most of them, including ZNF502, ZNF727, ZNF415, ZNF385B, ZNF232, ZNF200, and ZC3H14, have been validated as critical genes implicated in the pathogenesis of AD (Cassandri et al., 2017; Roubroeks et al., 2020; Vasanthakumar et al., 2020; Bu et al., 2021; Li et al., 2021). However, their roles in the molecular process of cognitive decline are not yet clear. We found that methylations of ZNF727 and ZNF502 have opposite effects on the progression of LMCI to AD. Consistent with this, previous studies have shown that the function of ZNFs could be distinct in altering cerebrospinal fluid (CSF) tau/ptau levels, promoting or inhibiting neuroinflammation in different regions, protecting or exposing neurons to oxidative stress–induced apoptosis, and interfering with the differentiation potential of neural stem cells (Cassandri et al., 2017; Calderari et al., 2018; Lopez et al., 2019; Baker et al., 2020; Bu et al., 2021). ZNFs act as transcription factors that modulate the expression of crucial genes involved in cellular biochemical processes by specifically binding to DNA or RNA (Farmiloe et al., 2020). Further studies of gene expression regulation related to these candidate ZNFs may be helpful to explore the onset and progression of cognitive impairment.

To the best of our knowledge, this is the first comprehensive genome-wide DNA methylation association analysis for LMCI. This analysis serves to elucidate the mechanisms of LMCI development, and aid in the prevention of LMCI progression to AD. However, due to the absence of relevant cellular biological experiments, the functionality of the noncoding gene RP11-526P5.2, which is associated with the top DMPs, could not be validated. It is important to mention another limitation of this study, namely, we only collected the methylation data from the ADNI cohort; thus, the results may be affected by the limitations imposed by the experimental design of the ADNI study. Therefore, it is imperative to expand the sample size and validate the experimental findings using datasets from other research centers to ensure the reliability of the results.

## Data Availability

Publicly available datasets were analyzed in this study. This data can be found here: ADNI datasets are publicly available (http://adni.loni.usc.edu/).
