# Supplementary material for: Epigenome-wide DNA methylation analysis of late-stage mild cognitive impairment
Source: Front Cell Dev Biol. 2024 Jan 16;12:1276288. doi: 10.3389/fcell.2024.1276288 (PMC10824854; doi:10.3389/fcell.2024.1276288)
Supplement: Supplementary file 1 [file DataSheet1.ZIP › Supplementary Material Presentation/Certificate of English language editing.pdf]

CERTIFICATE

OF ENGLISH LANGUAGE EDITING

Epigenome-wide DNA methylation analysis of late-stage mild cognitive impairment

Background: Patients with late-stage mild cognitive impairment (LMCI) have a higher risk of progression to Alzheimer's disease (AD) than those with early-stage mild cognitive impairment (EMCI). However, previous studies have often pooled EMCI and LMCI patients into a single MCI group, with limited independent investigation into the pathogenesis of LMCI.
Methods: In this study, we employed whole-genome methylation association analysis to determine the differences in peripheral blood methylation profiles between 663 CN and 554 LMCI patients.
Results: Our results revealed 2333 differentially methylated positions (DMPs) and 85 differentially methylated regions (DMRs) specific to LMCI. The top hit methylation sites or regions were associated with genes such as SNED1, RP11-526P5.2, histone deacetylases ...

This document certifies that the manuscript listed above was copy edited for English language by LetPub, with regard to grammar, punctuation, spelling, and clarity. Documents receiving this certification should be regarded as having undergone professional editorial revision for English language before submission. However, the authors may accept or reject LetPub's suggestions and changes at their own discretion and LetPub does not have editorial control over the submitted documents. Submitted documents may have new text that was not provided to LetPub for review. Please use the verification link below to determine the validity of the submitted version.

October 7, 2023

Date of Revision

LetPub

LetPub is an author service brand owned and operated by Accdon LLC.
Tel: 1-781-202-9968 Email: info@accdon.com
Address: 400 Fifth Ave, Suite 530, Waltham, MA 02451, United States

This manuscript has been individually edited for grammar, punctuation, spelling, and clarity. You may verify the authenticity of this certificate on our website (https://www.letpub.com/editorial-certificate) at any time using this manuscript's unique code: PRAA_231006H188FY.

© 2023 Accdon, LLC.
