# Supplementary material for: Epigenome-wide DNA methylation analysis of late-stage mild cognitive impairment
Source: Front Cell Dev Biol. 2024 Jan 16;12:1276288. doi: 10.3389/fcell.2024.1276288 (PMC10824854; doi:10.3389/fcell.2024.1276288)
Supplement: Supplementary file 1 [file DataSheet1.ZIP › Supplementary Material Presentation/Spplementary Figures/Supplementary Figure Legends.docx]

## Supplementary Figures

**Supplementary Figure 1.** Results of methylation data quality control. **(A)** distribution of beta value of methylation probs; **(B)** ratio of non-methylated to methylated sites.

**Supplementary Figure 2.** Quantile-quantile plot (QQ-plot) for epigenome-wide association analysis (EWAS). **(A)** QQ-plot of raw *p*-value with uniform distribution; **(B)** QQ-plot of t-value with Student's t-distribution.

**Supplementary Figure 3.** Volcano plot for epigenome-wide association analysis (EWAS).

**Supplementary Figure 4.** Late-stage mild cognitive impairment (LMCI) related differentially methylated probes (DMPs) enriched regions of genes RP11-526P5.2 **(A)** and TTC23 **(B)**. The most significant differentially methylated region (DMR) identified in this study that was associated with LMCI was annotated to RP11-526P5.2. Among the genomic regions annotated to protein-coding genes, the TTC23 locus exhibits the most pronounced concentration of significant CpG probes. Top panel: individual CpG association *p*-values; middle panel: gene structure (yellow strip: ENSEMBL genes; green strip: CGs island; red/brown strip: broad chromHMM; purple strip: regulation ENSEMBL; blue strip: SNP UCSC); bottom panel: pairwise correlation between CpG sites in this DMR.

**Supplementary Figure 5.** Late-stage mild cognitive impairment (LMCI) related differentially methylated probes (DMPs) enriched regions of HOXCs **(A)**, HOXDs **(B)**, ZENF727 **(C)** and ZNF502 **(D)**. Top panel: individual CpG association *p*-values; middle panel: gene structure (yellow strip: ENSEMBL genes; green strip: CGs island; red/brown strip: broad chromHMM; purple strip: regulation ENSEMBL; blue strip: SNP UCSC); bottom panel: pairwise correlation between CpG sites in this DMR.

**Supplementary Figure 6.** Late-stage mild cognitive impairment (LMCI) related differentially methylated probes (DMPs) enriched regions of HDAC4 **(A)** and HDAC6 **(B)**. Top panel: individual CpG association *p*-values; middle panel: gene structure (yellow strip: ENSEMBL genes; green strip: CGs island; red/brown strip: broad chromHMM; purple strip: regulation ENSEMBL; blue strip: SNP UCSC); bottom panel: pairwise correlation between CpG sites in this DMR.
