# Supplementary material for: Epigenome-wide DNA methylation analysis of late-stage mild cognitive impairment
Source: Front Cell Dev Biol. 2024 Jan 16;12:1276288. doi: 10.3389/fcell.2024.1276288 (PMC10824854; doi:10.3389/fcell.2024.1276288)
Supplement: Supplementary file 1 [file DataSheet1.ZIP › Supplementary Material Presentation/Supplementary Table6.docx]

**Supplementary Table 6.** The full list of differentially methylated probes (DMPs) related with ZNF family genes

| **Probes** | **Chr** | **Pos** | **Strand** | **GencodeCompV12** | **Beta** | **AveExpr** | **t** | ***P*-value** | **Adjusted *P*-value** |
| --- | --- | --- | --- | --- | --- | --- | --- | --- | --- |
| cg13947469 | Chr7 | 63505871 | - | ZNF727;RP11-3N2.13 | 12.95 | -1.43 | 6.36 | 2.78E^-10^ | 1.53E^-05^ |
| cg14768256 | Chr3 | 44754587 | + | ZNF502 | 10.27 | 1.06 | 5.90 | 4.82E^-09^ | 1.09E^-04^ |
| cg06088684 | Chr2 | 180610608 | - | ZNF385B | 7.71 | 2.79 | -5.41 | 7.44E^-08^ | 5.83E^-04^ |
| cg18831899 | Chr17 | 5019056 | - | ZNF232;USP6 | 7.28 | 2.03 | 5.33 | 1.17E^-07^ | 7.57E^-04^ |
| cg09560297 | Chr19 | 37406349 | - | ZNF568;ZNF829 | 6.22 | 0.94 | 5.11 | 3.65E^-07^ | 1.52E^-03^ |
| cg05769153 | Chr19 | 53636398 | + | ZNF415 | 5.86 | 1.75 | 5.04 | 5.36E^-07^ | 1.89E^-03^ |
| cg05223766 | Chr19 | 53590304 | + | ZNF160 | 5.02 | -2.07 | 4.86 | 1.33E^-06^ | 3.36E^-03^ |
| cg01511534 | Chr16 | 3284640 | - | ZNF200 | 4.73 | -1.72 | 4.80 | 1.81E^-06^ | 4.02E^-03^ |
| cg16428517 | Chr16 | 3317428 | + | ZNF263 | 4.35 | 2.51 | 4.71 | 2.75E^-06^ | 4.91E^-03^ |
| cg05241461 | Chr19 | 22816980 | - | ZNF492 | 4.32 | -3.48 | 4.71 | 2.82E^-06^ | 4.99E^-03^ |
| cg03060903 | Chr19 | 44813896 | - | ZNF235 | 3.37 | 2.03 | 4.49 | 7.89E^-06^ | 9.39E^-03^ |
| cg19791813 | Chr19 | 44555139 | - | ZNF223 | 3.03 | -2.04 | 4.41 | 1.15E^-05^ | 1.17E^-02^ |
| cg26535330 | Chr19 | 44953377 | - | ZNF229 | 2.71 | 2.23 | 4.33 | 1.62E^-05^ | 1.46E^-02^ |
| cg04060356 | Chr20 | 52226170 | - | ZNF217;AC005808.3 | 2.61 | -1.90 | 4.31 | 1.80E^-05^ | 1.56E^-02^ |
| cg21926094 | Chr17 | 5018984 | + | ZNF232;USP6 | 2.08 | 0.76 | 4.17 | 3.20E^-05^ | 2.17E^-02^ |
| cg01200051 | Chr7 | 55954647 | + | RP11-15K19.2;ZNF713 | 2.02 | -0.11 | -4.16 | 3.42E^-05^ | 2.23E^-02^ |
| cg06808011 | Chr16 | 3286070 | + | ZNF200 | 1.92 | 1.05 | 4.13 | 3.81E^-05^ | 2.39E^-02^ |
| cg26781129 | Chr3 | 44753946 | - | ZNF502 | 1.69 | -0.51 | 4.08 | 4.90E^-05^ | 2.78E^-02^ |
| cg07908290 | Chr5 | 150284927 | + | ZNF300 | 1.66 | -2.29 | 4.07 | 5.11E^-05^ | 2.86E^-02^ |
| cg14674574 | Chr19 | 44575832 | - | ZNF284 | 1.63 | 1.10 | 4.06 | 5.24E^-05^ | 2.89E^-02^ |
| cg25565608 | ChrX | 47930609 | + | ZNF630 | 1.41 | -1.44 | -4.00 | 6.65E^-05^ | 3.31E^-02^ |
| cg20862309 | Chr14 | 89029074 | - | ZC3H14 | 1.39 | -0.20 | 4.00 | 6.81E^-05^ | 3.35E^-02^ |
| cg14931645 | Chr1 | 249133278 | - | ZNF672 | 1.36 | -3.26 | 3.99 | 7.06E^-05^ | 3.43E^-02^ |
| cg08271804 | Chr19 | 22816896 | - | ZNF492 | 1.26 | -2.13 | 3.96 | 7.88E^-05^ | 3.63E^-02^ |
| cg18305433 | Chr6 | 28972963 | + | ZNF311 | 1.22 | 2.51 | 3.95 | 8.25E^-05^ | 3.72E^-02^ |
| cg22502448 | Chr3 | 44622831 | + | ZNF660;RP11-944L7.4 | 1.09 | 1.27 | -3.92 | 9.50E^-05^ | 4.02E^-02^ |
| cg05269323 | Chr7 | 150067712 | + | REPIN1;RP4-584D14.5;ZNF775 | 1.07 | 2.26 | 3.91 | 9.67E^-05^ | 4.04E^-02^ |
| cg19541622 | Chr4 | 56180 | - | ZNF595 | 1.07 | 0.49 | 3.91 | 9.68E^-05^ | 4.05E^-02^ |
| cg05801999 | Chr19 | 36737202 | + | ZNF565 | 0.91 | -0.20 | -3.87 | 1.16E^-04^ | 4.40E^-02^ |
| cg26482862 | Chr18 | 32957463 | - | ZNF396 | 0.84 | -0.46 | 3.85 | 1.25E^-04^ | 4.63E^-02^ |
| cg01919488 | chr2 | 71558589 | + | ZNF638 | 0.75 | -2.46 | 3.82 | 1.38E^-04^ | 4.92E^-02^ |
