# Supplementary figures and images for: Epigenome-wide DNA methylation analysis of late-stage mild cognitive impairment
Source: Front Cell Dev Biol. 2024 Jan 16;12:1276288. doi: 10.3389/fcell.2024.1276288 (PMC10824854; doi:10.3389/fcell.2024.1276288)

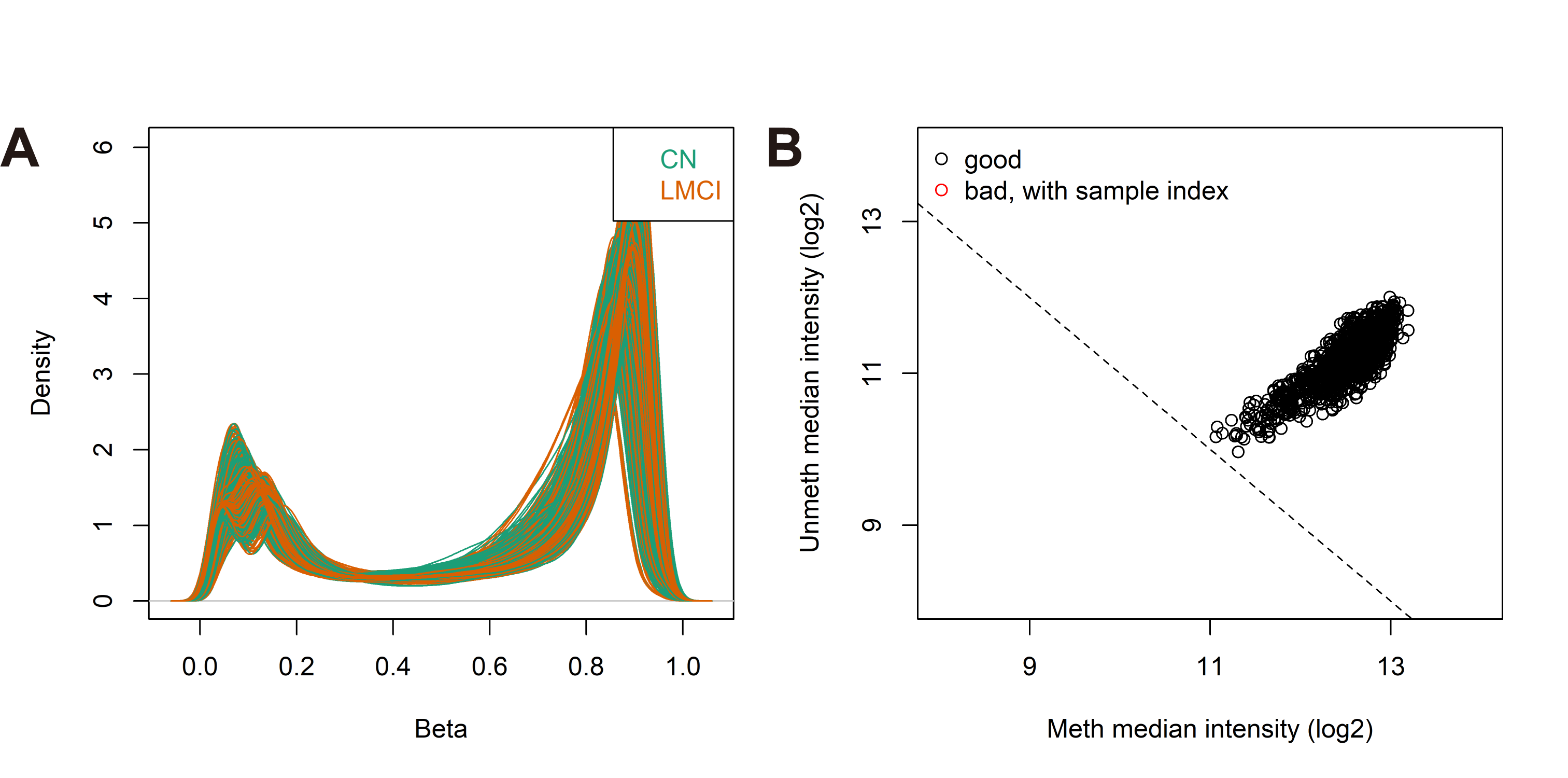

Supplement: Supplementary file 1 [file DataSheet1.ZIP › Supplementary Material Presentation/Spplementary Figures/Supplementary Figure 1.tiff]

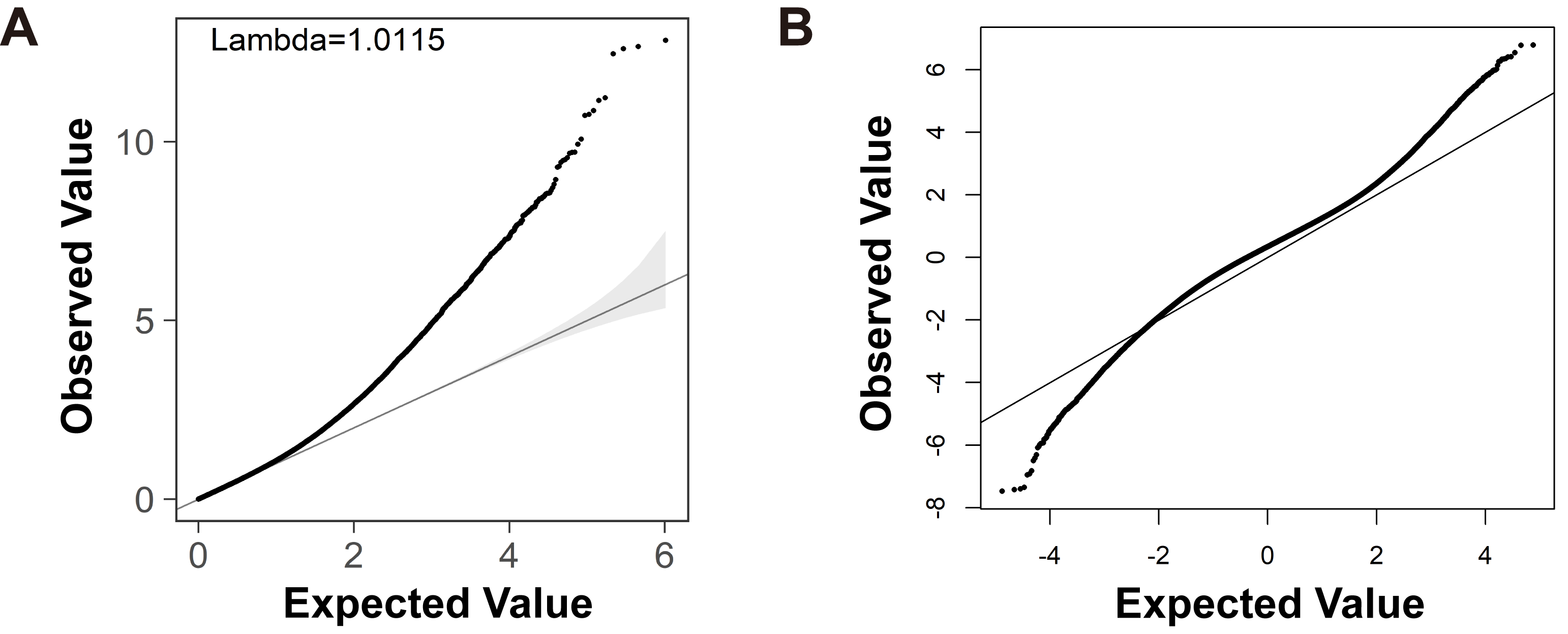

Supplement: Supplementary file 1 [file DataSheet1.ZIP › Supplementary Material Presentation/Spplementary Figures/Supplementary Figure 2.tiff]

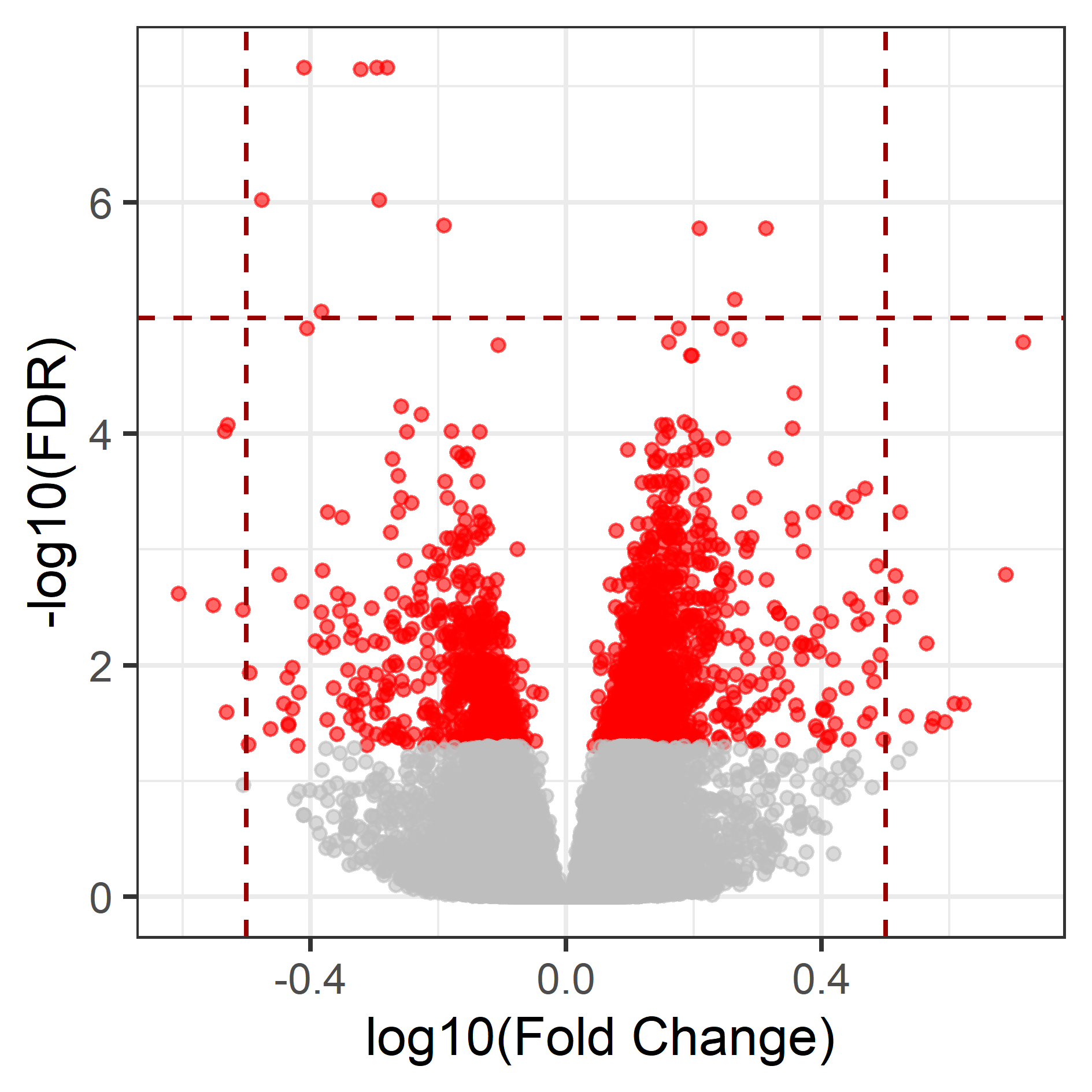

Supplement: Supplementary file 1 [file DataSheet1.ZIP › Supplementary Material Presentation/Spplementary Figures/Supplementary Figure 3.tiff]

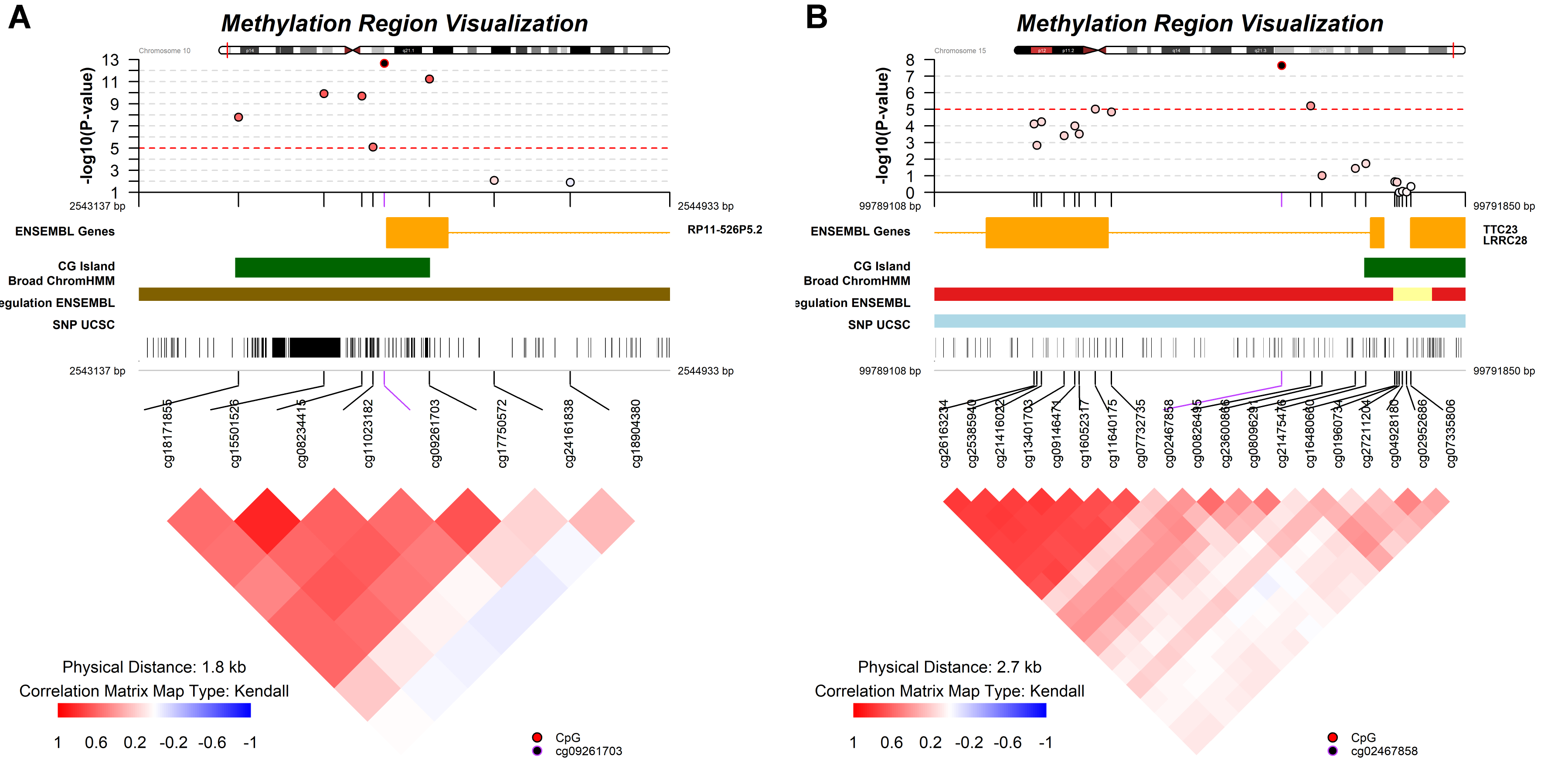

Supplement: Supplementary file 1 [file DataSheet1.ZIP › Supplementary Material Presentation/Spplementary Figures/Supplementary Figure 4.tiff]

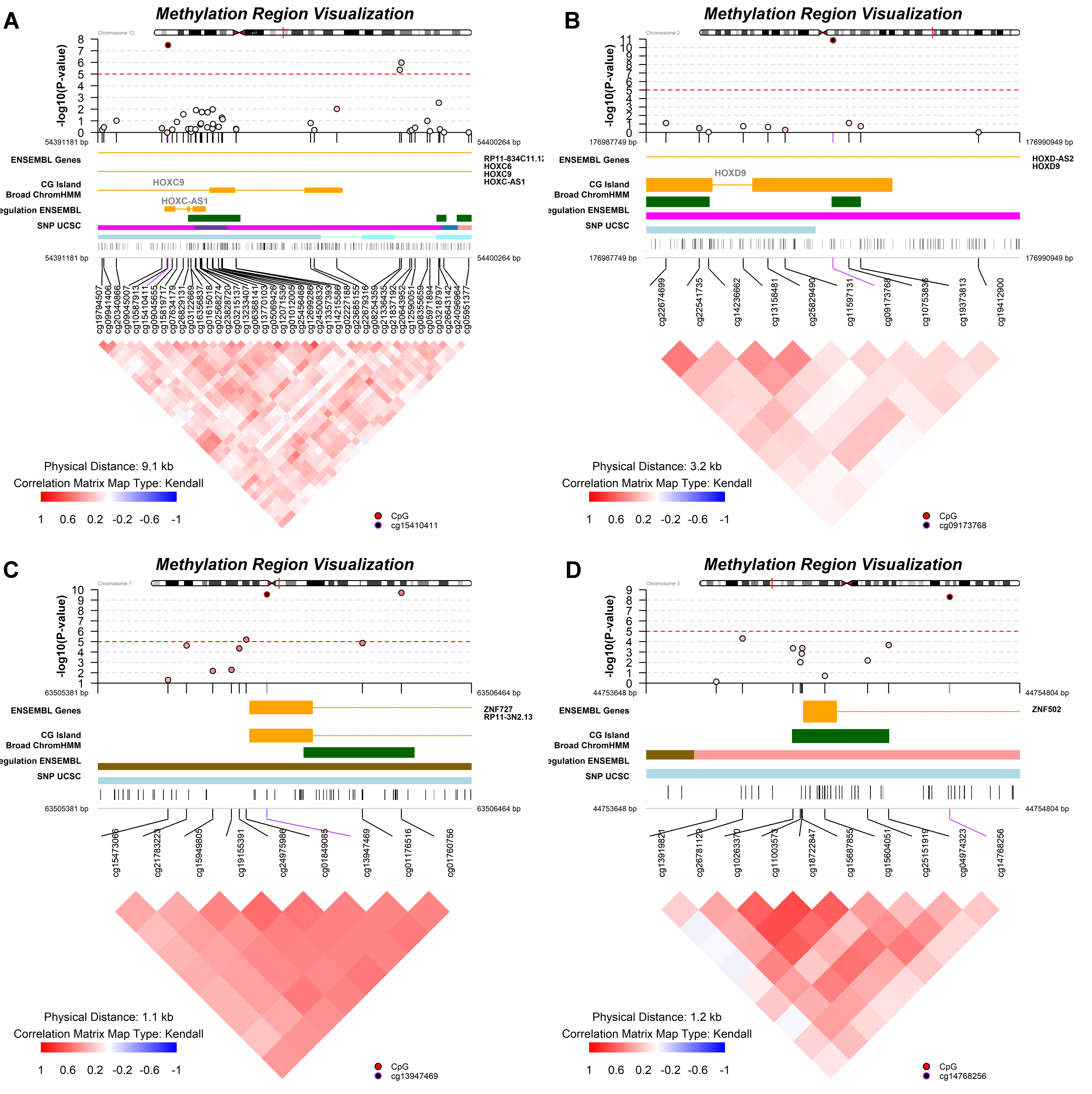

Supplement: Supplementary file 1 [file DataSheet1.ZIP › Supplementary Material Presentation/Spplementary Figures/Supplementary Figure 5.tiff]

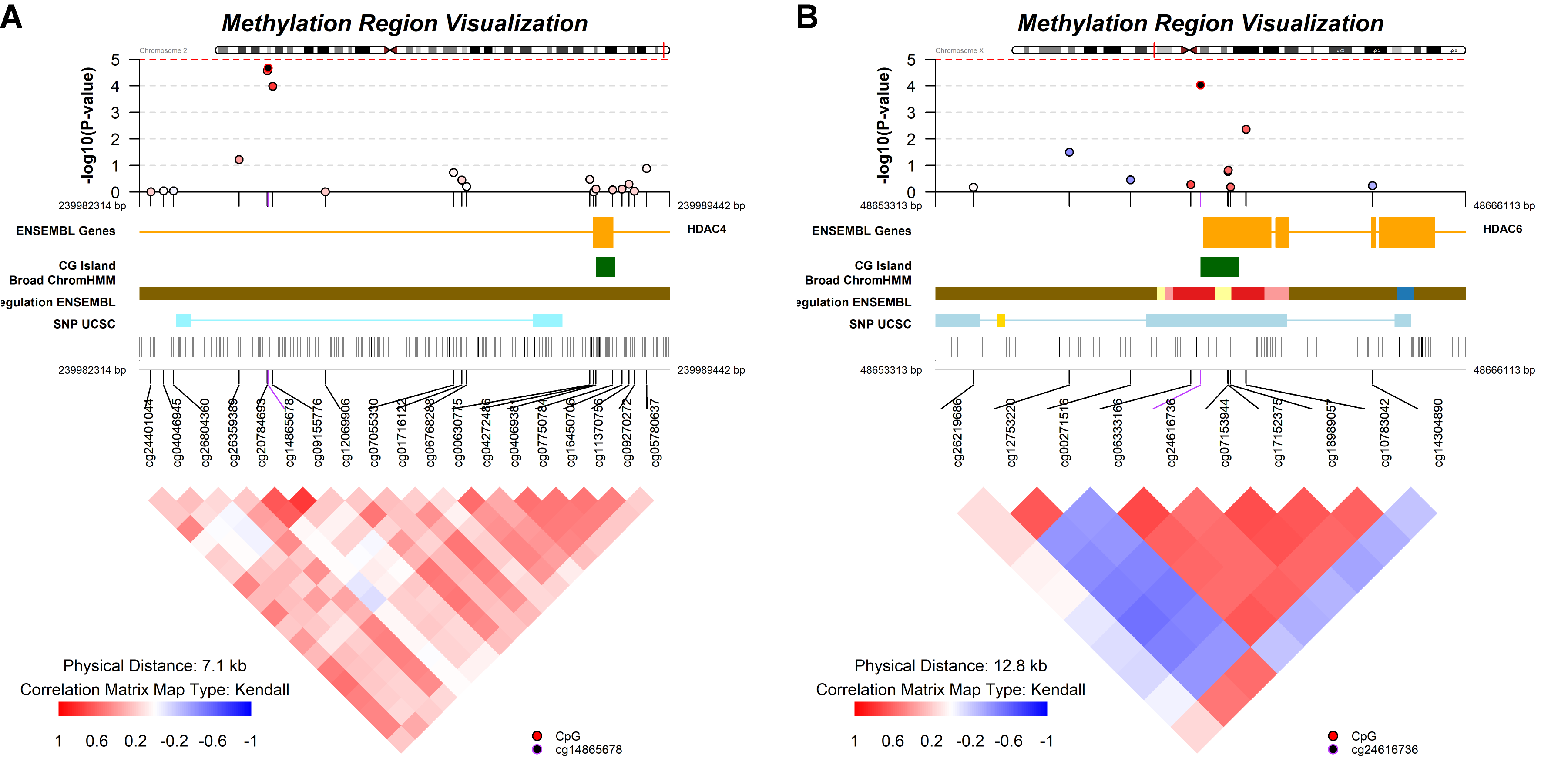

Supplement: Supplementary file 1 [file DataSheet1.ZIP › Supplementary Material Presentation/Spplementary Figures/Supplementary Figure 6.tiff]
